# *MoIVD*-Mediated Leucine Catabolism Is Required for Vegetative Growth, Conidiation and Full Virulence of the Rice Blast Fungus *Magnaporthe oryzae*

**DOI:** 10.3389/fmicb.2019.00444

**Published:** 2019-03-14

**Authors:** Ya Li, Xiuxia Zheng, Minghui Zhu, Mengting Chen, Shengnan Zhang, Fangyuan He, Xiaomin Chen, Jiarui Lv, Mengtian Pei, Ye Zhang, Yunhui Zhang, Wenzong Wang, Jing Zhang, Mo Wang, Zonghua Wang, Guangpu Li, Guodong Lu

**Affiliations:** ^1^State Key Laboratory of Ecological Pest Control for Fujian and Taiwan Crops, College of Plant Protection, Fujian Agriculture and Forestry University, Fuzhou, China; ^2^Key Laboratory of Ministry of Education for Genetics, Breeding and Multiple Utilization of Crops, Plant Immunity Center, Fujian Agriculture and Forestry University, Fuzhou, China; ^3^Institute of Oceanography, Minjiang University, Fuzhou, China; ^4^Department of Biochemistry and Molecular Biology, University of Oklahoma Health Sciences Center, Oklahoma City, OK, United States

**Keywords:** *Magnaporthe oryzae*, isovaleryl-CoA dehydrogenase, leucine catabolism, biological function, fungal development and pathogenesis

## Abstract

Isovaleryl-CoA dehydrogenase (IVD), a member of the acyl-CoA dehydrogenase (ACAD) family, is a key enzyme catalyzing the conversion of isovaleryl-CoA to β-methylcrotonyl-CoA in the third reaction of the leucine catabolism pathway and simultaneously transfers electrons to the electron-transferring flavoprotein (ETF) for ATP synthesis. We previously identified the ETF ortholog in rice blast fungus *Magnaporthe oryzae* (*MoETF*) and showed that *MoETF* was essential for fungal growth, conidiation and pathogenicity. To further investigate the biological function of electron-transferring proteins and clarify the role of leucine catabolism in growth and pathogenesis, we characterized *MoIVD* (*M. oryzae* isovaleryl-CoA dehydrogenase). MoIvd is highly conserved in fungi and its expression was highly induced by leucine. The Δ*moivd* mutants showed reduced growth, decreased conidiation and compromised pathogenicity, while the conidial germination and appressorial formation appeared normal. Consistent with a block in leucine degradation, the Δ*moivd* mutants accumulated isovaleric acid, grew more slowly, fully lacked pigmentation and completely failed to produce conidia on leucine-rich medium. These defects were largely rescued by raising the extracellular pH, suggesting that the accumulation of isovaleric acid contributes to the growth and conidiation defects. However, the reduced virulence of the mutants was probably due to their inability to overcome oxidative stress, since a large amount of ROS (reactive oxygen species) accumulated in infected host cell. In addition, MoIvd is localized to mitochondria and interacted with its electron receptor MoEtfb, the β subunit of MoEtf. Taken together, our results suggest that *MoIVD* functions in leucine catabolism and is required for the vegetative growth, conidiation and full virulence of *M. oryzae*, providing the first evidence for IVD-mediated leucine catabolism in the development and pathogenesis of plant fungal pathogens.

## Introduction

Rice blast caused by *Magnaporthe oryzae* is a devastating disease in rice growing areas across the world (Valent and Chumley, 1991; Tablot, 2003; Fernandez and Orth, 2018). Each year, this fungus results in an economic loss estimated to be $66 billion, which could feed 60 million people (Pennisi, 2010). The infection begins with three-celled conidia of *M. oryzae* contacting and germinating on the host surface, and then a dome-shaped infection structure called appressorium is developed from the germ tube end (Hamer et al., 1988). The mature appressoria melanize and accumulate high Turgor pressure (Howard et al., 1991), which assists the penetration peg in mechanically breaching the host cell wall (Kankanala et al., 2007). Once the fungus overcomes host defense response and completes colonization, the infectious hyphae, developed from penetration peg, grow intra- and intercellularly in the host and produce necrotic lesions within 3–5 days (Sakulkoo et al., 2018). Finally, new conidia are formed and released to start a new infection cycle. In addition to rice blast, wheat blast caused by *M. oryzae* has also recently emerged in South America and Bangladesh (Islam et al., 2016; Sadat and Choi, 2017). Due to its economic importance, genetic tractability and genome sequence availability, *M. oryzae* has emerged as a model organism to study the fungal pathogenesis and interaction with host plants (Dean et al., 2005; Ebbole, 2007). Understanding important metabolic processes in *M. oryzae* should help to develop novel and effective strategies to control this disease.

Leucine is one of the three branched amino acids, and its catabolism provides key intermediate metabolites—acetyl-CoA and ATP—for other metabolic processes (Lei et al., 2012; Arany and Neinast, 2018). Isovaleryl-CoA dehydrogenase (IVD) is an enzyme catalyzing the third reaction of the leucine catabolism pathway by conversion of isovaleryl-CoA to β-methylcrotonyl-CoA, and simultaneously electrons transfer to the respiratory chain for ATP production via electron-transferring flavoprotein (ETF) (Mohsen et al., 1998; Zhang et al., 2006; Mohsen and Vockley, 2015). As a flavoprotein with flavin adenine dinucleotide (FAD) as the cofactor, IVD catalyzes α,β-dehydrogenation and removal of one hydrogen as a proton from the branched-chain substrate, isovaleryl-CoA (Tiffany et al., 1997). Structurally, IVD is a member of the acyl-CoA dehydrogenase (ACAD) family and shares high sequence homology and similar mechanism with other family members for the α,β-dehydrogenation of acyl-CoA substrates (Rozen et al., 1994; Mohsen et al., 1998; Ghisla and Thorpe, 2004). According to the chain length and whether it branches, the ACAD family is divided into short/branched-chain acyl-CoA dehydrogenases (SBCAD), short-chain acyl-CoA dehydrogenases (SCAD), medium-chain acyl-CoA dehydrogenases (MCAD), long-chain acyl-CoA dehydrogenases (LCAD), and very long chain acyl-CoA dehydrogenases (VLCAD) (Ghisla and Thorpe, 2004). IVD belongs to the SBCAD subfamily that also includes isobutyryl-CoA dehydrogenase (IBD) and 2-methyl branched-chain acyl-CoA dehydrogenase (MBCAD), which are involved in the catabolism of valine and isoleucine, respectively (Noda et al., 1980; Brosnan and Brosnan, 2006).

Leucine intake and catabolism are important to humans, and the function of human IVD in leucine catabolism has been extensively reported (Vockley and Ensenauer, 2006). Individuals carrying IVD mutations are unable to break down leucine properly and display symptoms including poor feeding, vomiting, seizures, and even stupor (Vockley and Ensenauer, 2006; Kaya et al., 2013). Because isovaleric acid accumulates in the patient’s plasma, this disease is also called isovaleric acidemia (Tanaka, 1990; Pascarella et al., 2011; Erdem et al., 2010). A distinctive odor characterized as “sweaty feet” can be noted in body secretions during acute episodes (Tokatli et al., 1998). Plants, fungi and bacteria can synthesize leucine, unlike humans. However, the function of IVD-mediated leucine catabolism is not as well understood in those species as in humans. In plants, the identification, characterization and purification of pea IVD were reported, and this IVD was localized to the mitochondria and played a regulatory role in development (Däschner et al., 1999; Reinard et al., 2000). Arabidopsis IVD oxidizes catabolic intermediates of both leucine and valine, and also functions as alternative electron donor linking leucine catabolism to the electron transport chain for ATP production (Däschner et al., 2001; Araújo et al., 2010). Likewise, potato IVD is also involved in the catabolism of both leucine and valine (Faivre-Nitschke et al., 2001). In fungi, the only report of an IVD relates to glutamic acid-mediated repression of the promoter activity of *Aspergillus oryzae* IVD gene (Yamashita et al., 2007). In bacteria, *Micrococcus luteus* IVD is specific for leucine degradation, and the IVD-deficient mutant cannot grow on leucine (Surger et al., 2018).

The upstream leucine synthesis pathway in *M. oryzae* is required for conidiation, appressorium Turgor establishment and host penetration as demonstrated by characterizing two acetolactate synthase genes—*MoILV2* and *MoILV6*—and one threonine dehydratase gene—*MoILV1*—in the pathway (Du et al., 2013, 2014). But the downstream leucine catabolism pathway has not been characterized so far. In our previous study, *M. oryzae* electron-transferring flavoprotein ETF (*MoETF*) was functionally characterized and shown to be indispensable for fungal growth, conidiation and pathogenicity (Li et al., 2016). However, the function of its electron donors, acyl-CoA dehydrogenases, remains unknown. Here, we focus on one of the predicted ETF electron donors—*MoIVD* (*M. oryzae* Isovaleryl-CoA dehydrogenase gene id *MGG_02540*)—and demonstrate its biological function in the development and pathogenesis of *M. oryzae*.

## Materials and Methods

### Strains and Growth Conditions

The *M. oryzae* strain Guy11 strain was used as background to construct the Δ*moivd* mutants (Δ*moivd-3* and *-6*) and complementary strain (Δ*moivd-3/MoIVD*). The CM (complete medium) and MM (minimal medium) were prepared for testing the fungal growth and conidiation as described previously (Li et al., 2010a). Conidia were harvested from a fungal colony, growing for 12 days in a 9 cm plate, and the production was quantified by a hemacytometer. Conidia suspension was set in a concentration of 5 × 10^4^ conidia ml^-1^ and placed on a hydrophobic surface in a humid environment at 25°C to induce conidia germination and appressorium formation. *Escherichia coli* strain DH-5α was used for routine bacterial transformations and maintenance of various plasmid vectors. The culturing medium for DH-5α is liquid and agar LB (Luria-Bertani) medium. By trying a concentration gradient of 0.3 mM, 3 mM, 30 mM, 0.1 M and 1 M, we found that the optimal leucine concentration used in CM and MM to treat the fungus was 0.1 M and 3 mM, respectively. As described previously, 50 mM sodium acetate and sodium carbonate were added to CM to treat the fungus (Li et al., 2016).

### Molecular Manipulation

To construct the *MoIVD* deletion vector, the 1 kb up- and down-stream fragment from the *MoIVD* coding region were amplified and fused with the up- and down-half of the *HPH* (hygromycin phosphotransferase) gene, respectively, using overlapping PCR. The up- and down-half fragments of the *HPH* sequence shared an overlapping region of 100 bp in length. For building *MoIVD* complementation vectors, the full length *MoIVD* with a native promoter region (about 1.5-kb) was cloned to the pCB1532 vector. The 1.5 kb *GFP* sequence harboring a TrpC-terminator was inserted into the C-terminal of *MoIVD* to construct the MoIvd-GFP strain to explore the protein localization. qRT-PCR was performed by following the kit of “One Step qRT-PCR Kit RNA-direct Realtime PCR Master Mix” (Code Number QRT-101B). To do semi qRT-PCR, the RNA was isolated, and the cDNA was synthesized by using the kit “PrimeScript^TM^ 1st Strand cDNA Synthesis” (Code Number 6110A) prior to conventional PCR. For doing the Y2H (yeast two-hybrid) assay, *MoIVD* and *MoETFA/B* were cloned to pGBKT7 and pGADT7, respectively, and then the resulting plasmids were transformed to the AH109 yeast strain. The yeast clones were grown on -Leu/-Trp/-Ade/-His/SD (synthetical defined) medium to test whether the proteins interact. To do the BIFC (bimolecular fluorescence complementation) assay, *MoIVD* and *MoETFA/B* were fused to the N terminal and C terminal of YFP (Yellow Fluorescent Protein), respectively, to construct the MoIvd-NYFP-pKNT and MoEtfa/b-CYFP-pCX62 plasmids, and then the plasmids were transformed to Guy11 to analyze whether the whole YFP signal appears in the conidia of transformants. Primers used in this study are listed in Supplementary Table S1.

### Fungal Transformation

Fungal protoplast was prepared as described previously (Li et al., 2014). To perform gene deletion transformation, no less than 2 μg *MoIVD* deletion vector DNA was introduced to Guy11 protoplast, and transformants were selected for hygromycin resistance. Southern blotting was conducted to confirm the deletion events using the digoxigenin (DIG) high prime DNA labeling and detection starter Kit I (11745832910 Roche Germany). A 900 bp upstream fragment of *MoIVD* locus was selected as a probe for hybridization to digested genomes of all fungal strains. *MoIVD* complementation transformation was performed by introducing complementation vector DNA into mutant protoplast and screening transformants against chlorimuron-ethyl resistance.

### Conidial Induction and Infection Related Assay

To perform conidia induction assay, thin mycelial plugs were excised from colonies and placed on glass slides. After incubating 24 h with constant light, the conidia development state was observed and imaged by microscopy. The conidia suspension was prepared in a concentration of 5 × 10^4^ conidia ml^-1^ for infection-related assay. To perform the live rice infection assay, an airbrush was used and 5 ml conidial suspension was sprayed on 10 plants of 15-day-old dwarf Indica rice cv. CO-39. Post-spray, inoculated plants were kept in a sealed chamber with 90% relative humidity at 25°C for 24 h. Then, the inoculated plants were removed from the chamber to allow disease symptoms to develop over 4–5 days. The pathogenicity assay on barley was performed by dropping the conidial suspension on excised Golden Promise leaves in 20 μl drops (Li et al., 2010b). The disease spots on barley develop within 5–6 days. The live sheath assay was performed as described previously to evaluate invasive growth (Xu et al., 1998).

### Cytorrhysis Assay

The cytorrhysis assay was performed to test appressorium turgor as follows. Conidial suspension was prepared at a concentration of 5 × 10^4^ conidia ml^-1^ and 10 μl drops were placed on hydrophobic slides for 24 h at 25°C to allow appressoria maturation. Then the covering water was carefully removed and replaced with an equal volume of glycerol in concentrations of 2.0, 3.0, and 4.0 M. After continued incubation for 15 min, the ratio of collapsed appressoria was recorded in each glycerol treatment and to reflect the appressorium turgor difference between different fungal strains.

### DAB (3,3′-Diaminobenzidine) Staining

Host ROS accumulation during *M. oryzae* infection was detected by DAB staining assay as described previously (Chen et al., 2014). The conidial suspension was prepared at a concentration of 5 × 10^4^ conidia ml^-1^ and sprayed to barley leaves. After 24 h, the leaves were moved to 1 mg/ml DAB solution and kept at room temperature for 8 h for staining. Then, the samples were destained in washing solution (ethanol/acetic acid = 94/4, v/v) for 2–3 h. The ROS in the infected cell are meant to be detected by accumulation of a dark brown precipitate.

### Fatty Acids Content Test

The short chain fatty acids content was analyzed as follows. The 1.0 g dry mycelia samples were added to 3 ml water and vortexed with glass beads for 2 min. After centrifugation in the cold, the supernatant was transferred to a new tube and hydrochloric acid (1:10) was added to acidify the fat. Ether was added and the solution was mixed for 10 min for extraction. The ether phase sample was subjected to GC-MS (gas chromatography-mass spectrometer) analysis for short chain fatty acid analysis. For medium and long chain fatty acid testing, the sample was mixed with 2 ml 5% methanol hydrochloride, 3 ml chloroform methanol (1:1), 100 μl of methyl 19 alkylate internal standard and incubated in an 85°C water bath for 1 h. After cooling, 1 ml n-hexane was added for extraction with 2 min of shaking, and then the mixture was left standing for 1 h for stratification prior to GC-MS analysis.

### Amino Acids Content Test

Samples of 1.0 g dry mycelia were transferred to 20 ml hydrolysis tube, and 16 ml 6 mol/l HCL solutions was added to resuspend the mycelium. Samples were then degassed for 30 min, sealed by nitrogen and hydrolyzed at 110°C for 22–24 h. After cooling, samples were transferred to a volumetric flask to a final volume of 50 ml with deionized water. A 1 ml sample was transferred to a small bottle, deacidized and dried in a vacuum. The sample was recovered with 1 ml deionized water and dried twice, and 1 ml 0.02 M hydrochloric acid was added and dissolved fully. A 500 μl sample of the hydrolysate was placed in a 5 ml plastic centrifuge tube with 250 μl of 1 M triethylamine acetonitrile and mixed prior to addition of 25 μl of 0.1 M isothiocyanate phenyl acetonitrile. After mixing, the sample was placed at room temperature for 1 h, 2 ml of n-hexane was added, and the mixture was vortexed. After 10 min, the lower phase was collected and filtered through a 0.22-micron filter prior to HPLC (high performance liquid chromatography) analysis.

## Results

### MoIvd Is Highly Conserved in Fungi

**FIGURE 1:**
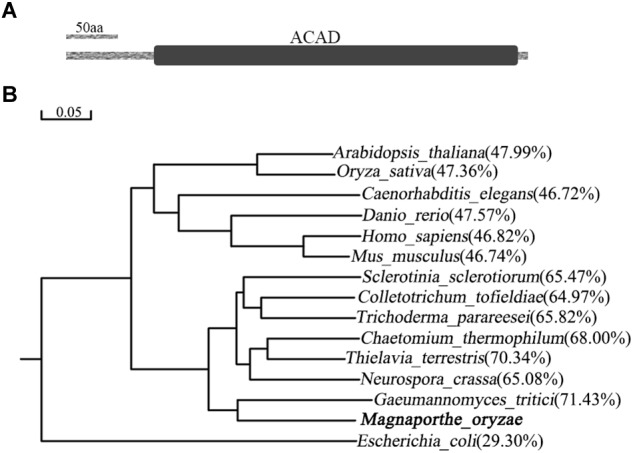
Domain structure of MoIvd and phylogenetic analysis of IVD homologs. **(A)** MoIvd domain prediction by the PFAM website (http://pfam.xfam.org/). The ACAD domain located between aa91 and aa464. The bar indicates 50 amino acids. **(B)** IVD phylogenetic tree constructed using the observed divergency method by DNAMAN6.0. The IVD protein sequences of 15 model species were collected from NCBI database. The bar indicates 0.05 distance units. The percentage in brackets indicates sequence identity between MoIvd and corresponding homolog. The sequence accession numbers are listed as follows: *Magnaporthe oryzae* (XP_003721261.1), *Arabidopsis thaliana* (NP_190116.1), *Oryza sativa* (XP_015639342.1), *Caenorhabditis elegans* (NP_500720.1), *Danio rerio* (NP_958899.1), *Homo sapiens* (NP_002216.2), *Mus musculus* (NP_062800.1), *Sclerotinia sclerotiorum* (APA11203.1), *Colletotrichum tofieldiae* (KZL76390.1), *Trichoderma parareesei* (OTA08745.1), *Chaetomium thermophilum* (XP_006691847.1), *Thielavia terrestris* (XP_003651516.1), *Neurospora crassa* (XP_964284.1), *Gaeumannomyces tritici* (XP_009227932.1) and *Escherichia coli* (WP_001189854.1).

*MoIVD* is located between +2434073 and +2436238 on chromosome 7 of *M. oryzae* genome in the 70–15 genome sequence (NCBI assembly GCA_000002495.2). The gene encodes a 470-amino acid (aa) protein including a long ACAD domain located between aa91 and aa464 (Figure 1A). The amino acid sequence of MoIvd was used to search for homologs in the NCBI database by BLAST analysis^1^. As a result, more than 100 MoIvd homologs with over 70% sequence identity were found from different fungal species (Supplementary Figure S1), suggesting that MoIvd is highly conserved in fungi. With the IVD homologs from several model species, we constructed a phylogenetic tree to analyze their evolutionary relationship. As shown in Figure 1B, MoIvd is more evolutionarily related to fungal homologs of *Gaeumannomyces tritici*, *Neurospora crassa*, *Thielavia terrestris*, *Colletotrichum tofieldiae* and *Sclerotinia sclerotiorum*, with 65–70% sequence identity. The plant and animal homologs *Arabidopsis thaliana*, *Oryza sativa*, *Caenorhabditis elegans*, *Danio rerio*, *Homo sapiens* and *Mus musculus* are more distant relatives from MoIvd with only 46–48% sequence identity. The bacterial homolog, *Escherichia coli*, is on the most distant evolutionary branch, with 30% sequence identity to MoIvd.

### *MoIVD* Expression Is Highly Induced by Exogenous Leucine

**FIGURE 2:**
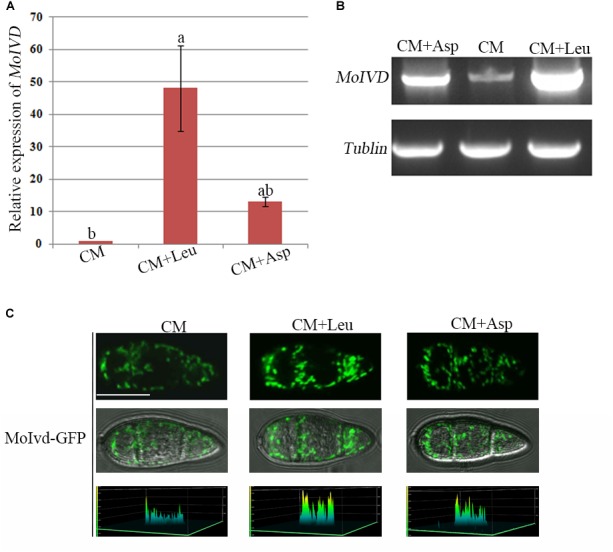
The *MoIVD* expression was highly induced by exogenous leucine. **(A)** qRT-PCR test of the relative *MoIVD* expression in fungus treated with 0.1 M leucine (Leu) and 0.1 M aspartic acid (Asp). **(B)** Semi qRT-PCR and electrophoresis test of *MoIVD* expression in fungus treated with 0.1 M Leu and 0.1 M Asp. **(C)** The fluorescence intensity of MoIvd-GFP in conidia treated with 0.1 M Leu and 0.1 M Asp. The peaks in the 3D chart show the signal intensity. The 3D chart was generated by laser scanning confocal microscopy. Bar = 10 μm.

To determine if *MoIVD* is involved in leucine catabolism, we investigated the *MoIVD* expression level under exogenous leucine stress via the approaches of qRT-RCR and semi qRT-RCR. *M. oryzae* was cultured in liquid CM containing 0.1 M exogenous leucine or in control CM without leucine or with 0.1 M asparagine, for 48 h at 25°C. In the presence of exogenous leucine, the *MoIVD* expression was highly induced, and its mRNA level increased by 50-fold and 5-fold, respectively, compared to the CM and the asparagine controls (Figure 2A). The stronger band of the *MoIVD* cDNA fragment in the leucine treatment also suggested a high mRNA level of *MoIVD* (Figure 2B).

We also constructed a MoIvd-GFP strain under the control of *MoIVD* promoter and determined the *MoIVD* expression level in the cell by confocal fluorescence microscopy. As shown in Figure 2C, MoIvd-GFP conidia displayed much brighter fluorescence in leucine treatment than in controls, suggesting higher expression levels of MoIvd-GFP in the presence of exogenous leucine. These results are consistent with a previous report that the promoter activity of IVD gene in *Aspergillus oryzae* was highly induced by the hydrophobic amino acid L-leucine, slightly induced by L-asparagine with low hydrophobicity, but not induced by L-glutamic acid sodium salt without hydrophobicity (Yamashita et al., 2007).

### *MoIVD* Is Involved in Vegetative Growth and Conidiation

**FIGURE 3:**
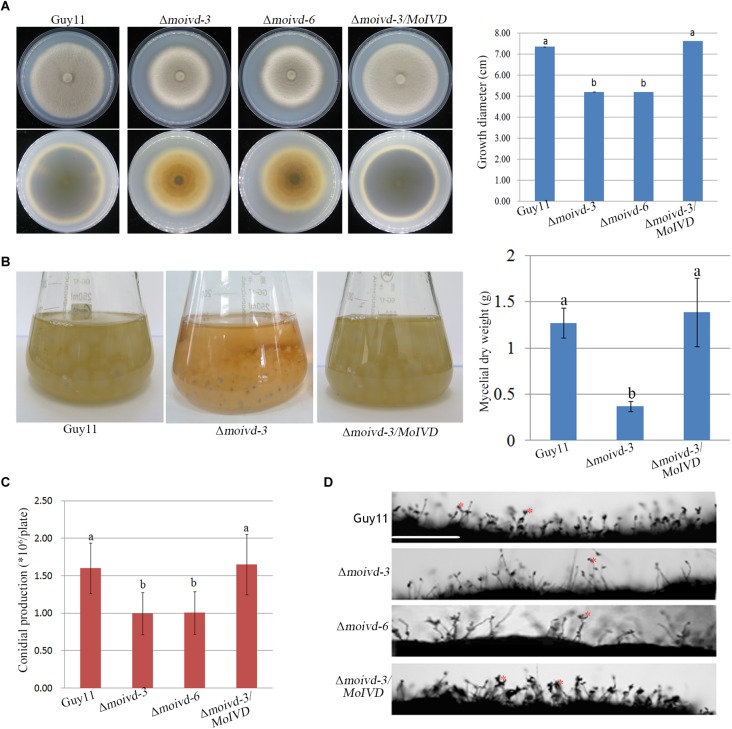
The growth and conidiation phenotype of the Δ*moivd* mutants. **(A)** The colonial morphology of the Δ*moivd* mutants growing on complete medium for 10 days. The upper left panel is the colony outlook. The lower left panel is the opposite side of culture plate. The bar chart in the right shows the growth size of the Δ*moivd* mutants. **(B)** The culture morphogenesis of Δ*moivd-3* incubated in liquid CM for 48h in flask (left) and bar chart showing the dry weight of Δ*moivd-3* culture (right). **(C)** Bar chart showing the conidial production of the Δ*moivd* mutants by culture on complete medium for 12 days. **(D)** Microscopic observation of the sporulating structures of the Δ*moivd* mutants. ^∗^ indicates conidia developed from conidiophores. Bar = 50 μm. All the data in this figure were calculated from three independent replicates. Lowercase ‘a’ or ‘b’ on the sample bar indicate no significant differences between samples. The different lowercase letters indicate significant differences (*P* < 0.05; *t* test).

To investigate the function of *MoIVD* in *M. oryzae*, we constructed two Δ*moivd* mutants (Δ*moivd-3* and Δ*moivd-6*) and one complementation strain Δ*moivd-3/MoIVD*. All the mutant and complemented strains were confirmed by Southern blot analysis (Supplementary Figure S2). Their growth phenotype was determined by the size and color of fungal colonies on CM medium. Our data indicated that the colony size of the Δ*moivd* mutants was smaller and the edge of mutant colonies also lost pigmentation in comparison to the controls, Guy11 and Δ*moivd-3/MoIVD* (Figure 3A). In this regard, the opposites of plates inoculated with the mutant strains completely lost color, while those of Guy11 and Δ*moivd-3/MoIVD* exhibited the normal black color (Figure 3A). Moreover, when cultured in liquid CM, the mutants produced a very small number of mycelia, and its dry weight was significantly reduced compared to Guy11 and Δ*moivd-3/MoIVD* (Figure 3B). These results indicated multiple growth defects in the Δ*moivd* mutants. We also examined conidiation in the mutants. The conidia were harvested from colonies growing on CM plates for 12 days and quantified using a hemacytometer. Our results showed that Δ*moivd-3* and Δ*moivd-6* produced fewer conidia (1 × 10^6^ spores per plate), which was about 2/3 of the conidia produced by strains Guy11 or Δ*moivd-3/MoIVD* (Figure 3C). Furthermore, the mutant conidiophores also decreased compared to those of Guy11 and Δ*moivd-3/MoIVD* (Figure 3D). Taken together, the data suggested that *MoIVD* is involved in vegetative growth and conidiation in *M. oryzae*.

### *MoIVD* Is Required for *M. oryzae* Pathogenicity

**FIGURE 4:**
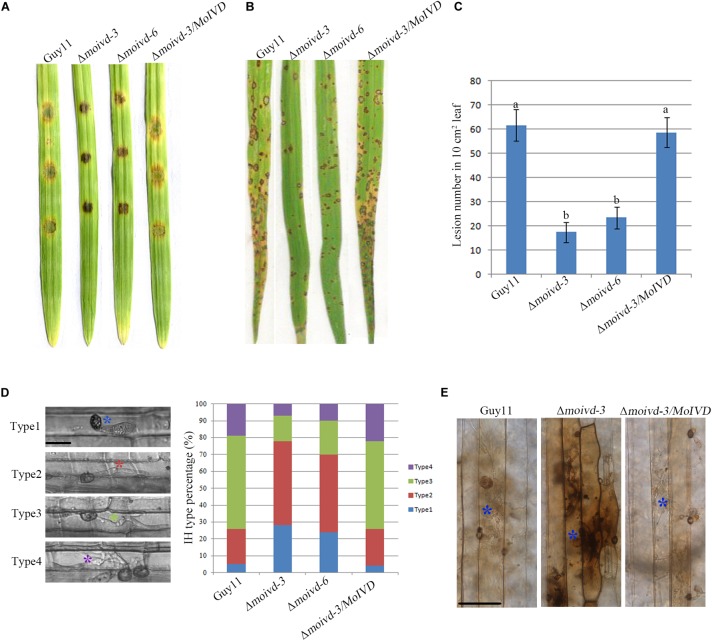
The pathogenicity assay of the Δ*moivd* mutants. **(A)** Pathogenicity test of the Δ*moivd* mutants on excised barley leaves. The conidium was set at a concentration of 1 × 10^5^ spores/ml. **(B)** Spray inoculation assay of the Δ*moivd* mutants on rice seedlings. The conidium was set at a concentration of 5 × 10^4^ spores/ml. **(C)** Quantitative analysis of lesion numbers in 10 cm^2^ leaf size. The data was calculated from three independent replicates. The same lowercases ‘a’ or ‘b’ on the sample bar indicate no significant differences between samples. The different lowercases indicate significant differences (*P* < 0.05; *t* test). **(D)** Statistic analysis of the invasive hyphae growth rate. The hyphae are divided into four types according to the growth speed. Type 1 indicates no penetration; Type 2 indicates penetration with a small peg; Type 3 represents the invasive hyphae in extension. Type 4 indicates the hyphae crossing the second host cell. Different color asterisk indicates the different hyphae type. Bar = 10 μm. **(E)** DAB staining of barley leaves infected by Δ*moivd-3*after inoculation for 24 h. The asterisk indicates the stained infected host cell by DAB (1 mg/ml). Bar = 50 μm.

To investigate the role of *MoIVD* in *M. oryzae* pathogenicity, we evaluated the mutant phenotype in infection-related development including conidial germination, appressorium formation and host infection. Although we did not find any significant difference between mutant and wild type strains in conidial germination and appressorium formation (Supplementary Table S2), their virulence on excised barley leaves showed differences, and Δ*moivd-3* and Δ*moivd-6* produced smaller disease lesions than Guy11 and Δ*moivd-3/MoIVD* (Figure 4A). Likewise, the mutants also produced much smaller and fewer lesions on rice seedlings than Guy11 and Δ*moivd-3/MoIVD* (Figure 4B,C). Furthermore, sheath infection assay was used to examine infectious growth in host cells, and Δ*moivd-3* and Δ*moivd-6* displayed lower penetration frequency and delayed hyphal growth after penetration in comparison to Guy11 and Δ*moivd-3/MoIVD* at 24 h post-inoculation/infection (Figure 4D). These results suggest *MoIVD* is required for full pathogenicity of *M. oryzae*. ROS accumulation is a symbol of host defense against pathogen attack and could be tested by DAB staining assay. Using this method, we found that the barley cells infected by the mutant exhibited a darker brown color than the ones infected by Guy11 and complemented strain Δ*moivd-3/MoIVD* (Figure 4E), suggesting more ROS accumulation in the mutant-infected cells.

### The Δ*moivd* Mutants Cannot Utilize Exogenous Leucine Properly

**FIGURE 5:**
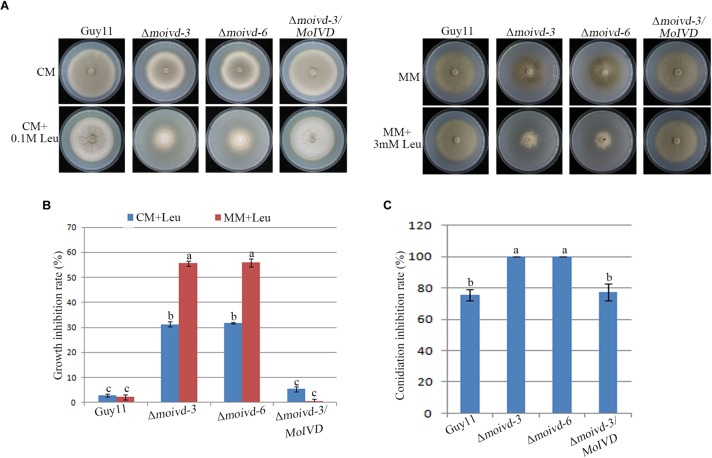
The growth and conidiation phenotype of the Δ*moivd* mutants under leucine treatment. **(A)** The growth phenotype of the Δ*moivd* mutants growing on complete medium (CM) and minimal medium (MM) with adding leucine (Leu). The left panel is the assay performed on CM medium. The right represents MM. The upper panel represents the colony front. The lower panel represents the back of the culture plate. As the mutant growth on MM was very sensitive to high concentrations of leucine, only 3 mM leucine was added in MM. **(B)** Bar chart showing the growth inhibition rate of the Δ*moivd* mutants growing on leucine containing medium. **(C)** Bar chart showing the conidiation inhibition rate of the Δ*moivd* mutants growing on leucine containing CM medium. All these data were calculated from three independent replicates. The same lowercases ‘a’ or ‘b’ on the sample bar indicate no significant differences between samples. The different lowercases indicate significant differences (*P* < 0.05; *t* test).

To further investigate whether *MoIVD* functions in leucine catabolism and whether *MoIVD* deletion affects the leucine degradation in *M. oryzae*, we evaluated the ability of utilizing exogenous leucine by the Δ*moivd* mutants. By adding extra leucine to the CM and MM media and testing the effect on fungal growth, we found that the Δ*moivd-3* and Δ*moivd-6* grew much slower and their colonies lost pigmentation completely in contrast to those growing on normal CM or MM medium (Figure 5A,B), suggesting a defect in degradation of exogenous leucine. Furthermore, we found that the mutant could not produce any conidia when growing on leucine-containing medium, although Guy11 and Δ*moivd-3/MoIVD* also reduced conidia production to some extent (Figure 5C). Unlike leucine, two other branched-chain amino acids, isoleucine and valine, had no effect on the growth and conidiation of the mutants (Supplementary Figure S3). These results indicated that *MoIVD* is essential for leucine degradation and further confirmed *MoIVD* function in the leucine catabolism pathway.

### Isovaleric Acid Accumulation Is Responsible for the Mutant Growth and Conidiation Defects

**FIGURE 6:**
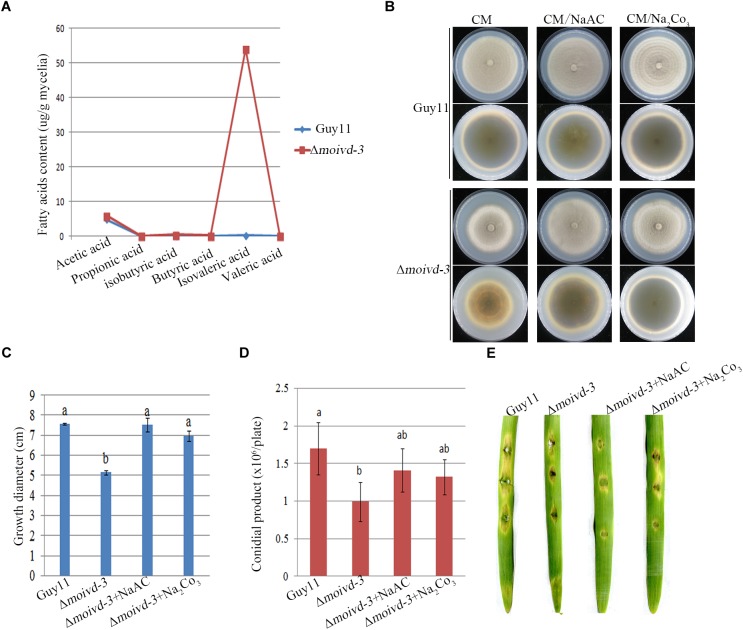
Isovaleric acid accumulation in Δ*moivd-3* and the effect of exogenous NaAc and Na_2_Co_3_ to mutant phenotype. **(A)** Line chart showing 6 short chain fatty acid content tested by GCMS. **(B)** The growth phenotype of Δ*moivd-3* growing on NaAc and Na_2_Co_3_ treated CM medium. **(C)** Bar chart showing the growth size of Δ*moivd-3* growing on NaAc and Na_2_Co_3_ treated CM medium. **(D)** Bar chart showing the conidial product of Δ*moivd-3* growing on NaAc and Na_2_Co_3_ treated CM medium. **(E)** No effect of NaAc or Na_2_Co_3_ on the virulence of Δ*moivd-3* on excised barley leaves. All the data were calculated from three independent replicates. The same lowercases ‘a’ or ‘b’ on the sample bar indicate no significant differences between samples. The different lowercases indicate significant differences (*P* < 0.05; *t* test).

When growing on CM plates, the Δ*moivd* mutants released an unpleasant odor, which is consistent with a previous report of smelly odor produced by accumulation of isovaleric acid in blood samples of human patients with IVD deficiency (Tokatli et al., 1998). To this end, we performed GC-MS to test the content of 6 short chain fatty acids in Δ*moivd-3*. Our results showed a very high concentration of isovaleric acid (54.06 ± 5.55 μg/g) in Δ*moivd-3*, which was about 450-fold that in Guy11 (0.12 ± 0.03 μg/g) (Figure 6A and Supplementary Figure S4). As a control, we also examined the content of 35 medium and long chain fatty acids by GC-MS and found no significant difference between Δ*moivd-3* and Guy11 except several fatty acids with slight changes (Supplementary Figure S5). Thus, *MoIVD* deletion specifically resulted in isovaleric acid accumulation.

To determine if the accumulation of isovaleric acid contributed to the defects of growth and conidiation in the Δ*moivd* mutants, we used the weak alkaline of sodium acetate to treat Δ*moivd-3* and hoped to neutralize the excessive acid in the mutant. Indeed, Δ*moivd-3* almost fully recovered the growth rate and colony color by addition of 50 mM sodium acetate to the medium (Figure 6B,C), and conidia production was also largely restored to the Guy11 level (Figure 6D). Like sodium acetate, sodium carbonate was also able to rescue mutant growth and conidiation (Figure 6B–D). However, both sodium acetate and sodium carbonate were unable to rescue the mutant pathogenicity (Figure 6E).

### *MoIvd* Is Localized in Mitochondria

**FIGURE 7:**
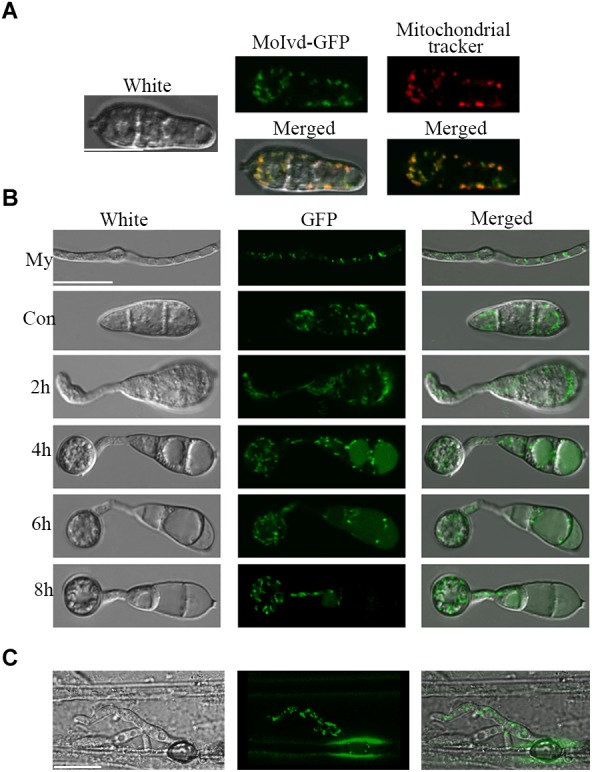
Subcellular localization of MoIvd-GFP. **(A)** MoIvd-GFP co-localizes with the mitochondrial tracker showing red light analyzed by laser scanning confocal microscopy. The tracker was purchased by Invitrogen with catalog number M7152. Image was taken after the conidia were treated with tracker for 3 min. Bar = 10 μm. **(B)** MoIvd-GFP stably expressed during mycelia (My) growth, conidia (Con) and appressorium development. Bar = 10 μm. **(C)** MoIvd-GFP expressed in invasive hyphae by barley infection assay. Bar = 10 μm.

To further clarify the function of *MoIVD*, we determined the intracellular localization of MoIvd-GFP in complemented strain Δ*moivd-3/MoIVD* by confocal microscopy. Our results showed that the green signal of MoIvd-GFP fully co-localized with the red signal of mitochondrial tracker in the conidia, suggesting that MoIvd is localized in the mitochondria (Figure 7A). We further demonstrated the mitochondrial localization of MoIvd-GFP in all developmental stages, including mycelium, conidia, conidial germination, appressoria formation and invasive hyphae (Figure 7B,C). We conclude that MoIvd functions in mitochondria throughout the entire life cycle of *M. oryzae*.

### MoIvd Interacts With MoEtfb, the Electron-Transferring Flavoprotein β Subunit in *M. oryzae*

**FIGURE 8:**
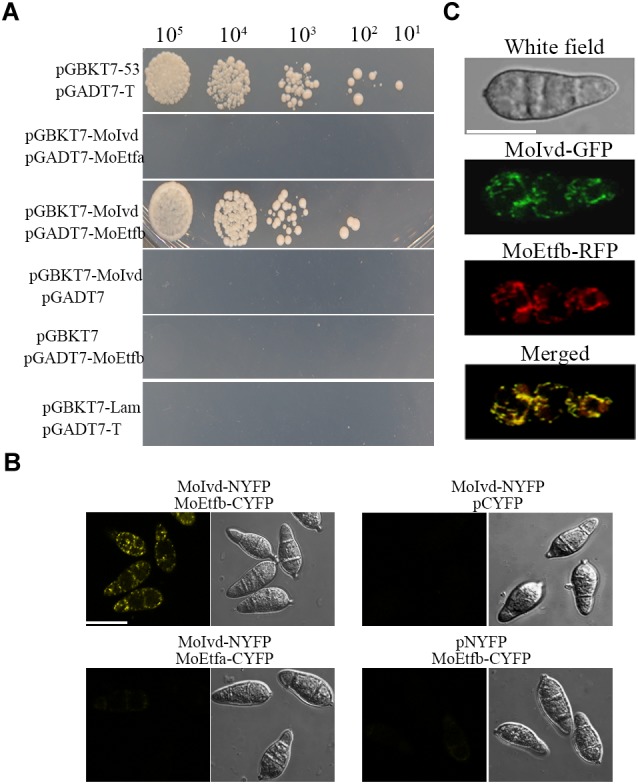
MoIvd interacted and co-localized with MoEtfb. **(A)** Yeast two hybrids showing that MoIvd interacted with MoEtfb, but not MoEtfa. The yeast transformants were grown on -His/-Ade/-Trp/-Leu/SD medium with 2 mM 3AT to test the interaction. **(B)** BIFC showing that MoIvd interacted with MoEtfb by displaying whole yellow signal in conidia transformed with MoIvd-NYFP and MoEtfb-CYFP. Bar = 10 μm. **(C)** MoIvd-GFP was co-localized with MoEtfb-RFP by showing overlapping GFP and RFP fluorescence in conidia transformed with MoIvd-GFP and MoEtfb-RFP. Bar = 10 μm.

Our previous study identified the MoEtf, which consists of two subunits—MoEtfa and MoEtfb—as a key regulator of fungal growth, conidiation and pathogenictiy in *M. oryzae* (Li et al., 2016), and IVD was predicated as one of the ETF electron donors in human (Watmough and Frerman, 2010). To clarify whether there is any interaction between the two proteins in *M. oryzae*, we conducted Y2H and BIFC assays. As shown in Figure 8A, MoIvd was able to interact with MoEtfb but not MoEtfa in the Y2H assay. In the BIFC assay, MoIvd and MoEtf were fused to the N- and C- terminus of YFP, respectively, and then co-transformed into *M. oryzae*. Our results showed the interaction fluorescent signal in the conidia co-expressing MoIvd and MoEtfb, but not in the negative controls, and also showed the conidia expressing MoIvd and MoEtfa (Figure 8B). Furthermore, we demonstrated co-localization of MoIvd-GFP and MoEtfb-RFP in the mitochondria of conidia (Figure 8C). These results suggested that MoIvd and MoEtf may function in the same complex for transferring electrons in the mitochondria of *M. oryzae*.

## Discussion

In this study, we show that *MoIVD* deletion in *M. oryzae* resulted in reduced growth, decreased conidiation and compromised pathogenicity of *MoIVD*-defective strains. The mutant accumulated with a high level of isovaleric acid, and exogenous sodium acetate or sodium carbonate could rescue the mutant growth and conidiation defects (Figure 6B–D). To our knowledge, this is the first report that IVD-mediated leucine catabolism is essential to the development and pathogenesis of plant pathogens. As reported, the human IVD mutation resulted in the isovaleryl-CoA not being degraded normally in the body, where the by product isovaleric acid accumulated and yielded a sweaty foot smell (Tokatli et al., 1998; Erdem et al., 2010; Pascarella et al., 2011). In our study, the Δ*moivd* mutants also displayed a similar odor and excessive accumulation of isovaleric acid was detected. However, unlike the human case (Fries et al., 1996), the administration of glycine and L-carnitine in *M. oryzae* could not rescue the defects of Δ*moivd-3* (Supplementary Figures S6A,B). It is unclear if the alkalinization of the environment that we used to suppress the mutant defect could be applied to human IVD mutation.

Electron-transferring flavoprotein is predicated as the electron receptor of IVD. Our previous study functionally characterized the *MoETF* but did not investigate its association with *MoIVD* (Li et al., 2016). In this study, by a series of protein interaction assays (Figure 8A–C), we confirmed that MoIvd could constitute a complex with MoEtf by interacting with the β subunit of MoEtf (MoEtfb) for possible electron transferring. By further comparing the mutant phenotype, we found both mutants showed defects in vegetative growth, conidiation and virulence, but those in the Δ*moetf* mutant were more severe than those in the Δ*moivd* mutant strain. For instance, the Δ*moetf* mutant fully unpigemented, failed to produce conidia and completely lost virulence (Li et al., 2016). We suggest this is because *MoETF* functions as electron receptor of 8 predicted acyl-CoA dehydrogenases (gene id MGG_16316, MGG_03418, MGG_05949, MGG_15041, MGG_08690, MGG_08661, MGG_06561, and MGG_02540) in *M. oryzae*, and *MoETF* may play a much more extensive and crucial role than a single partner, acyl-CoA dehydrogenase gene (*MoIVD*). The function of other 7 acyl-CoA dehydrogenases in *M. oryzae* will be of interest in this regard. Another similar phenotype trait was that the Δ*moetf* mutant also released an unpleasant odor, probably from accumulated butyrate, and the mutant growth and conidiation, but not pathogenicity, could also be largely recovered by exogenous sodium acetate or carbonate (Li et al., 2016). These data indicate that, as electron transferring proteins, both *MoIVD* and *MoETF* are involved in vegetative growth, conidiation and pathogenicity, and the accumulated acid metabolites in the mutants specifically contribute to the growth and conidiation defects.

One of the important products of leucine metabolism is acetyl-CoA, but no report has shown that an IVD mutation in leucine metabolism could cause a shortage of acetyl-CoA and thereby lead to defects. This is likely because leucine catabolism is not the only pathway to produce acetyl-CoA (Lei et al., 2012) and other metabolic pathways would also have a compensation mechanism. Therefore, the byproducts caused by IVD mutation, such as isovaleric acid and hydroxyisovalerate (Erdem et al., 2010; Pascarella et al., 2011), would play more important roles in leading to mutant defects. Also confirmed by our study in *M. oryzae*, supplementation of acetyl-CoA could not rescue Δ*moivd-3* defects (Supplementary Figure S6C). This finding further suggests that the major reason responsible for the Δ*moivd* mutants’ defects is the excess isovaleric acid. However, the mutant defects in virulence were not caused by isovaleric acid, as they were not responsive to sodium acetate or carbonate (Figure 6E). Considering that the mutant could produce normal appressorium with enough turgor pressure for penetration (Supplementary Figure S7), we speculated that the reduced virulence of the mutant was more likely the result of not coping with oxidative stress during initial colonization. In our study, we confirmed this hypothesis by the result that ROS production was enhanced significantly during mutant-mediated infection (Figure 5E). To analyze whether the ROS production was induced by isovaleric acid, we treated barley leaves with 0.1, 1 and 10% isovaleric acid *in vitro* and then stained the leaves with DAB. Similar to the untreated leaves, no ROS was stained. This suggests that it is not isovaleric acid that induced high ROS production in the mutant infected host cells (Supplementary Figure S8). Given that *MoIVD* is involved in electron transferring and electron transferring dysfunction probably caused an imbalance of redox state (Lämmer et al., 2011), we speculate that the mutant defect in managing oxidative stress possibly results from imbalance of redox state, although more evidence is needed.

The IVD mutation destroys leucine catabolism at the third step, involving the conversion of isovaleryl-CoA to β-methylcrotonyl-CoA. An accumulation of leucine has not been observed in IVD mutants (Mohsen et al., 1998; Mohsen and Vockley, 2015) and we also did not find an obvious high level of leucine in Δ*moivd-3* (Supplementary Figure S9), but rather a high level of isovaleric acid was present (Figure 6A and Supplementary Figure S4). These findings suggest that the IVD mutation led to a block of leucine catabolism at the third step, and that accumulated isovaleryl-CoA is converted into isovaleric acid that is not further degraded. This explains why the Δ*moivd* mutants displayed a more severe defect in growth and conidiation when leucine was added to the culture medium (Figure 5). Addition of two other branched amino acids, valine and isoleucine, to the growth medium caused no obvious change in the growth and conidiation characteristics displayed by the mutant strain (Supplementary Figure S3). This result further suggested that *MoIVD* is specific for leucine catabolism in *M. oryzae*, which is not similar to the potato IVD, which has an additional role in degrading valine (Faivre-Nitschke et al., 2001).

**FIGURE 9:**
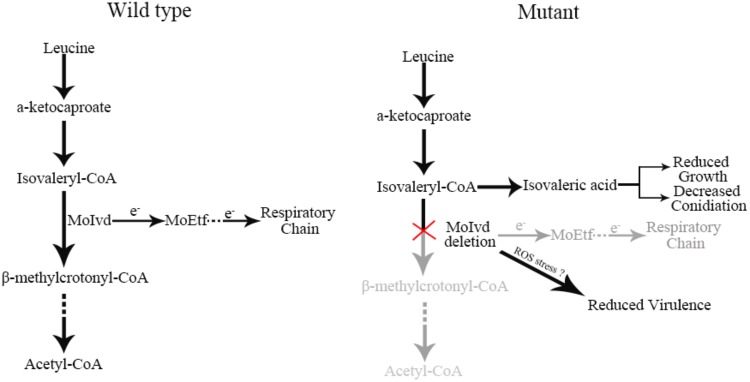
Proposed working model of *MoIVD* in *M. oryzae*. In wild-type strain, leucine is normally degraded by the leucine catabolism pathway and electrons are transferred to the respiratory chain from MoIvd, while in the Δ*moivd* mutants, the pathway was blocked at the third step where isovaleryl-CoA was converted into isovaleric acid. The isovaleric acid accumulation in mutant resulted in the reduced growth and decreased conidiation. The reduced virulence probably resulted from the mutant’s inability to overcome host ROS defense. “e^-^” indicates electron. “?” means a probable virulence reduction mechanism of mutant not coping with ROS stress during infection.

In summary, we functionally characterized MoIvd-mediated leucine catabolism in *M. oryzae* and proposed a working model in which the leucine-inducible MoIvd functions specifically in the third reaction of leucine catabolism in mitochondria (Figure 9). Upon *MoIVD* deletion, the isovaleryl-CoA could not be degraded and is converted to isovaleric acid, which accumulates in the mutant, possibly within mitochondria. The isovaleric acid accumulation at least partially contributed to the mutant growth and conidiation defects due to intracellular acidification, as those phenotypes were largely recovered by alkalinization of the medium. As the electron donor of *MoETF*, *MoIVD* deletion would block the MoIvd-MoEtf-mediated electron transfer reaction and probably result in an imbalance of redox state, which explains why the mutant could not overcome oxidative stress during infection and subsequently displayed a reduced virulence.

## Author Contributions

YL and GDL designed the experiments. YL and XZ analyzed the mutant phenotypes, processed the data and wrote the manuscript. MC, MZ, YHZ, WW, XC, and SZ constructed the gene deletion and complementation and collected the mutant phenotype data. FH, JL, MP, and YZ completed the protein localization experiments. JZ did the DAB staining assay. GPL, MW, and ZW revised the manuscript. All the authors approved the manuscript.

## Conflict of Interest Statement

The authors declare that the research was conducted in the absence of any commercial or financial relationships that could be construed as a potential conflict of interest.

## Footnotes

1 https://blast.ncbi.nlm.nih.gov/Blast.cgi
