# Supplementary material for: MoIVD-Mediated Leucine Catabolism Is Required for Vegetative Growth, Conidiation and Full Virulence of the Rice Blast Fungus Magnaporthe oryzae
Source: Front Microbiol. 2019 Mar 14;10:444. doi: 10.3389/fmicb.2019.00444 (PMC6426774; doi:10.3389/fmicb.2019.00444)
Supplement: Supplementary file 1 [file Data_Sheet_1.PDF]

Supplementary Material

MoIVD-mediated Leucine Catabolism is Required for Vegetative Growth, Conidiation and Full Virulence of the Rice Blast Fungus *Magnaporthe oryzae*

Ya Li[#]*, Xiuxia Zheng[#], Minghui Zhu, Mengting Chen, Shengnan Zhang, Fangyuan He, Xiaomin Chen, Jiarui Lv, Mengtian Pei, Ye Zhang, Yunhui Zhang, Wenzong Wang, Jing Zhang, Mo Wang, Zonghua Wang, Guangpu Li and Guodong Lu*.

[#]These authors contributed equally: Ya Li & Xiuxia Zheng

*Correspondence:
Dr. Ya Li & Dr. Guodong Lu
liya-81@163.com & gdlufafu@163.com

Supplementary Figures

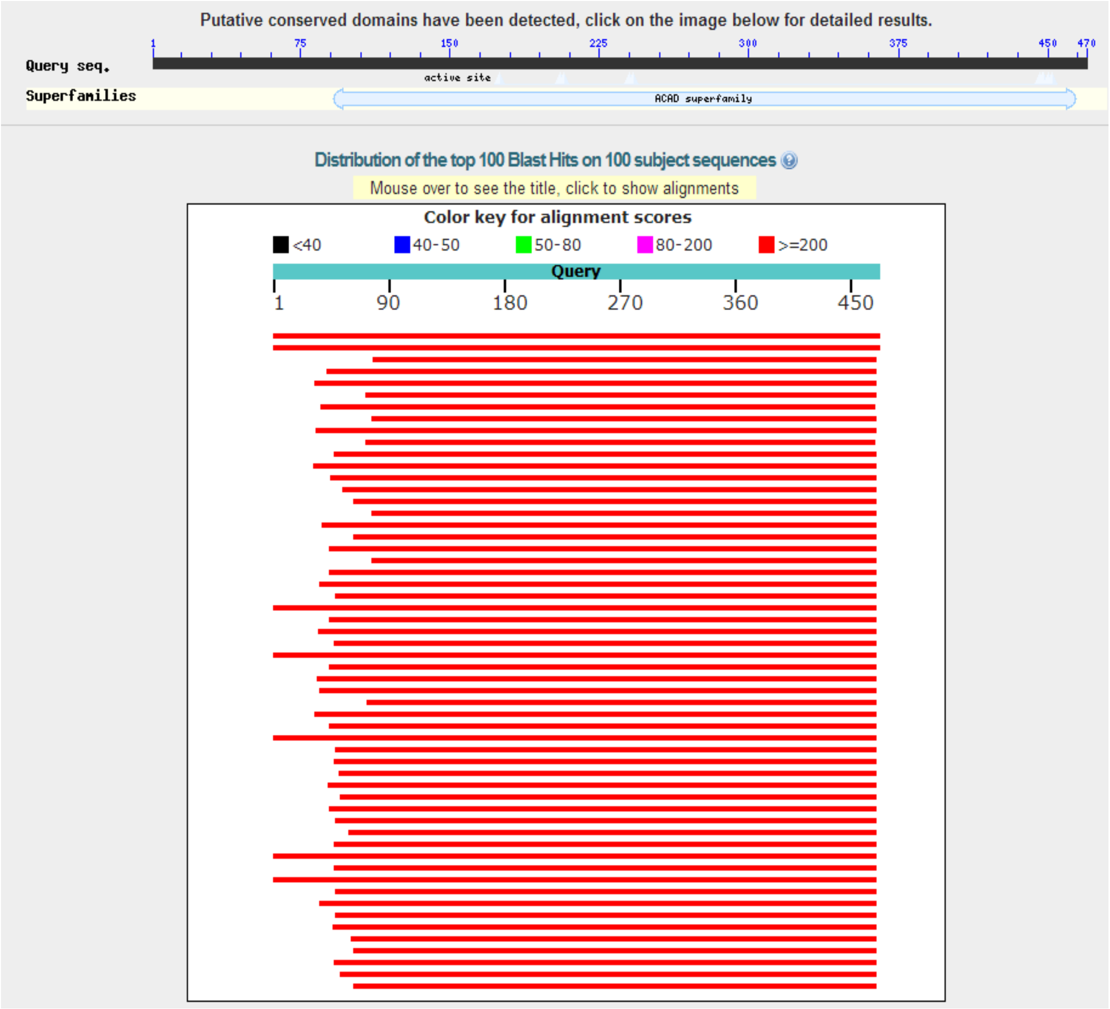

Sequences producing significant alignments:

Select: All None Selected:0

Alignments Download GenPept Graphics Distance tree of results Multiple alignment

Description	Max score	Total score	Query cover	E value	Ident	Accession
isovaleryl-CoA dehydrogenase 2 [Magnaporthe oryzae 70-15]	959	959	100%	0.0	100%	XP_003721261.1
isovaleryl-CoA dehydrogenase-like protein [Magnaporthe grisea]	952	952	100%	0.0	99%	AAX07730.1
hypothetical protein GGTG_11777 [Gaeumannomyces tritici R3-111a-1]	676	676	82%	0.0	82%	XP_009227932.1
hypothetical protein THITE_2111925 [Thielavia terrestris NRRL 8126]	640	640	90%	0.0	76%	XP_003651516.1
isovaleryl-CoA dehydrogenase-like protein [Chaetomium thermophilum var. thermophilum DSM 1495]	640	640	92%	0.0	73%	XP_006691847.1
hypothetical protein MYCTH_2297849 [Thermothelomyces thermophila ATCC 42464]	638	638	84%	0.0	78%	XP_003660054.1
conserved hypothetical protein [Chaetomium globosum CBS 148.51]	632	632	91%	0.0	72%	XP_001222512.1
hypothetical protein K445DRAFT_318236 [Daldinia sp. EC12]	631	631	83%	0.0	78%	OTB15395.1
uncharacterized protein PODANS_1_20100 [Podospora anserina S mat+]	631	631	92%	0.0	72%	XP_001907474.1
Isovaleryl-CoA dehydrogenase, mitochondrial [Madurella mycetomatis]	631	631	84%	0.0	77%	KXX80887.1
Acyl-CoA dehydrogenase NM [Glarea lozoyensis ATCC 20868]	630	630	89%	0.0	73%	XP_008076263.1
uncharacterized protein SMAC_05027 [Sordaria macrospora k-hell]	629	629	92%	0.0	70%	XP_003352913.1
acyl-CoA dehydrogenase NM domain-like protein [Cadophora sp. DSE1049]	627	627	90%	0.0	72%	PVH76285.1
isovaleryl-CoA dehydrogenase, mitochondrial precursor [Pochonia chlamydosporia 170]	627	627	87%	0.0	73%	XP_018145310.1
Isovaleryl-CoA dehydrogenase 1, mitochondrial [Scedosporium apiospermum]	627	627	86%	0.0	75%	XP_016639061.1
isovaleryl-CoA dehydrogenase [Neurospora crassa OR74A]	627	627	83%	0.0	76%	XP_964284.1
acyl-CoA dehydrogenase/oxidase [Microdochium bolleyi]	625	625	91%	0.0	72%	KXJ93923.1
hypothetical protein jhhlp_004038 [Lomentospora prolificans]	625	625	86%	0.0	74%	PKS09423.1
hypothetical protein FNYG_14496 [Fusarium nygamai]	625	625	90%	0.0	72%	PNP60736.1
hypothetical protein M426DRAFT_325299 [Hypoxylon sp. CI-4A]	625	625	83%	0.0	77%	OTA99259.1
probable isovaleryl-CoA dehydrogenase [Fusarium mangiferae]	625	625	90%	0.0	72%	CVK90758.1
isovaleryl-CoA dehydrogenase [Verticillium dahliae VdLs.17]	624	624	91%	0.0	71%	XP_009656246.1
acyl-CoA dehydrogenase [Colletotrichum orchidophilum]	624	624	89%	0.0	73%	XP_022473620.1
isovaleryl-CoA dehydrogenase [Hypoxylon sp. EC38]	624	624	99%	0.0	67%	OTA62724.1
probable isovaleryl-CoA dehydrogenase [Fusarium proliferatum]	624	624	90%	0.0	72%	CVL00118.1
isovaleryl-CoA dehydrogenase [Diaporthe helianthi]	624	624	91%	0.0	71%	POS81321.1
Acyl-CoA dehydrogenase/oxidase [Metarhizium majus ARSEF 297]	624	624	89%	0.0	72%	KIE00232.1
hypothetical protein ANO14919_058580 [fungal sp. No.14919]	623	623	99%	0.0	68%	GAW16431.1
probable isovaleryl-CoA dehydrogenase [Fusarium proliferatum ET1]	623	623	90%	0.0	72%	CZR42176.1
isovaleryl-CoA dehydrogenase (acyl-CoA dehydrogenase) [Colletotrichum tofieldiae]	623	623	92%	0.0	70%	KZL76390.1
isovaleryl-CoA dehydrogenase [Verticillium alfalfae VaMs.102]	623	623	91%	0.0	71%	XP_003007442.1
isovaleryl-CoA dehydrogenase mitochondrial precursor [Neurospora tetrasperma FGSC 2509]	623	623	84%	0.0	75%	EGZ78392.1
hypothetical protein sscle_07g059730 [Sclerotinia sclerotiorum 1980 UF-70]	622	622	92%	0.0	71%	APA11203.1
probable isovaleryl-CoA dehydrogenase [Fusarium fujikuroi]	622	622	90%	0.0	72%	SCO49226.1
IVD, Isovaleryl-CoA dehydrogenase [Trichoderma parareesei]	622	622	99%	0.0	66%	OTA08745.1
acyl-CoA dehydrogenase [Colletotrichum salicis]	622	622	89%	0.0	72%	KXH69216.1
Acyl-CoA dehydrogenase/oxidase [Metarhizium brunneum ARSEF 3297]	622	622	89%	0.0	72%	XP_014541815.1
predicted protein [[Nectria] haematococca mpVI 77-13-4]	622	622	88%	0.0	72%	XP_003052548.1
acyl-CoA dehydrogenase NM domain-like protein [Pezoloma ericae]	621	621	90%	0.0	71%	PMD14227.1
hypothetical protein M434DRAFT_395900 [Hypoxylon sp. CO27-5]	621	621	88%	0.0	72%	OTA93198.1
probable isovaleryl-CoA dehydrogenase [Fusarium fujikuroi IMI 58289]	621	621	90%	0.0	72%	XP_023432140.1
hypothetical protein VD0004_g6506 [Verticillium dahliae]	621	621	89%	0.0	72%	PNH40513.1
isovaleryl-CoA dehydrogenase [Fusarium verticillioides 7600]	621	621	87%	0.0	74%	XP_018745503.1
putative isovaleryl-CoA dehydrogenase IvdA [Metarhizium anisopliae]	621	621	89%	0.0	72%	KFG79992.1
IVD, Isovaleryl-CoA dehydrogenase [Trichoderma guizhouense]	620	620	99%	0.0	66%	OPB35938.1
Isovaleryl-CoA dehydrogenase [Metarhizium anisopliae BRIP 53293]	620	620	89%	0.0	72%	KJK75747.1
hypothetical protein M431DRAFT_524241 [Trichoderma harzianum CBS 226.95]	620	620	99%	0.0	66%	XP_024769393.1
isovaleryl-CoA dehydrogenase [Coniochaeta ligniaria NRRL 30616]	620	620	89%	0.0	72%	OIW31327.1
hypothetical protein BN1708_000030 [Verticillium longisporum]	620	620	91%	0.0	70%	CRJ79500.1
acyl-CoA dehydrogenase [Colletotrichum fioriniae PJ7]	620	620	89%	0.0	72%	EXF81490.1
hypothetical protein B2J93_335 [Marssonina coronariae]	619	619	89%	0.0	70%	OWP00479.1
isovaleryl-CoA dehydrogenase, mitochondrial precursor [Rhynchosporium agropyri]	619	619	86%	0.0	74%	CZS91797.1
hypothetical protein FAVG1_04335 [Fusarium avenaceum]	619	619	86%	0.0	74%	KIL91931.1
hypothetical protein V502_09311 [Pseudogymnoascus sp. VKM F-4520 (FW-2644)]	619	619	89%	0.0	73%	KFZ08497.1

acyl-CoA dehydrogenase [Metarhizium robertsii]	619	619	88%	0.0	73%	EXV00594.1
hypothetical protein FPSE_05771 [Fusarium pseudograminearum CS3096]	619	619	86%	0.0	74%	XP_009257164.1
Isovaleryl-CoA dehydrogenase [Trichoderma reesei QM6a]	619	619	85%	0.0	74%	XP_006966535.1
Acyl-CoA dehydrogenase/oxidase [Cordyceps confragosa RCEF 1005]	619	619	99%	0.0	66%	OAA75343.1
acyl-CoA dehydrogenase [Colletotrichum nymphaeae SA-01]	619	619	89%	0.0	73%	KXH49875.1
Acyl-CoA dehydrogenase [Colletotrichum higginsianum IMI 349063]	619	619	89%	0.0	72%	XP_018157176.1
acyl-CoA dehydrogenase NM domain-like protein [Phialocephala scopiformis]	619	619	84%	0.0	74%	XP_018064206.1
hypothetical protein V500_03449 [Pseudogymnoascus sp. VKM F-4518 (FW-2643)]	619	619	89%	0.0	73%	KFY94037.1
isovaleryl- dehydrogenase [Colletotrichum gloeosporioides Nara gc5]	619	619	89%	0.0	72%	ELA36522.1
isovaleryl-CoA dehydrogenase, mitochondrial precursor [Rhynchosporium secalis]	618	618	84%	0.0	75%	CZT48327.1
acyl- dehydrogenase [Colletotrichum incanum]	618	618	91%	0.0	70%	KZL86490.1
isovaleryl-CoA dehydrogenase [Trichoderma harzianum]	618	618	99%	0.0	66%	KKO99487.1
hypothetical protein CGLO_05131 [Colletotrichum gloeosporioides Cg-14]	618	618	89%	0.0	72%	EQB54981.1
hypothetical protein FPOA_04172 [Fusarium poae]	618	618	86%	0.0	74%	OBS23623.1
Isovaleryl-CoA dehydrogenase, mitochondrial [Neonectria ditissima]	618	618	88%	0.0	72%	KPM38800.1
Isovaleryl-CoA dehydrogenase [Escovopsis weberi]	618	618	99%	0.0	68%	KOS20053.1
hypothetical protein V494_03419 [Pseudogymnoascus sp. VKM F-4513 (FW-928)]	618	618	89%	0.0	72%	KFY40619.1
hypothetical protein M430DRAFT_47099 [Amorphotheca resinae ATCC 22711]	617	617	84%	0.0	75%	XP_024725563.1
isovaleryl- dehydrogenase protein [Rutstroemia sp. NJR-2017a WRK4]	617	617	85%	0.0	74%	PQE33627.1
isovaleryl- dehydrogenase protein [Rutstroemia sp. NJR-2017a BBW]	617	617	88%	0.0	71%	PQE15746.1
Isovaleryl-CoA dehydrogenase, mitochondrial [Valsa mali var. pyri]	617	617	87%	0.0	73%	KUI56092.1
hypothetical protein BN1723_004063 [Verticillium longisporum]	617	617	91%	0.0	70%	CRK34425.1
putative isovaleryl- dehydrogenase protein [Phaeoacremonium minimum UCRPA7]	617	617	84%	0.0	75%	XP_007915630.1
acyl-CoA dehydrogenase domain-containing protein [Marssonina brunnea f. sp. 'multigermtubi' MB_m1	617	617	87%	0.0	72%	XP_007297235.1
unnamed protein product [Fusarium venenatum]	617	617	86%	0.0	74%	CEI60682.1
acyl-CoA dehydrogenase NM domain-like protein [Meliniomyces variabilis F]	617	617	84%	0.0	74%	PMD30718.1
isovaleryl-dehydrogenase [Colletotrichum incanum]	617	617	84%	0.0	74%	PMD30718.1
isovaleryl- dehydrogenase [Fusarium langsethiae]	617	617	87%	0.0	73%	OHX00636.1
isovaleryl-CoA dehydrogenase [Sclerotinia borealis F-4128]	617	617	86%	0.0	73%	KPA42183.1
isovaleryl-CoA dehydrogenase [Fusarium graminearum PH-1]	617	617	84%	0.0	75%	ESZ97456.1
acyl-CoA dehydrogenase [Colletotrichum simmondsii]	616	616	86%	0.0	74%	XP_011320250.1
isovaleryl-dehydrogenase [Colletotrichum orbiculare MAFF 240422]	616	616	89%	0.0	72%	KXH46955.1
isovaleryl-CoA dehydrogenase, mitochondrial precursor [Phialocephala subalpina]	616	616	88%	0.0	72%	ENH86865.1
isovaleryl-CoA dehydrogenase, mitochondrial precursor [Rhynchosporium commune]	616	616	84%	0.0	74%	CZR63832.1
hypothetical protein LLEC1_05746 [Cordyceps confragosa]	616	616	86%	0.0	73%	CZS98031.1
Acyl-CoA dehydrogenase/oxidase [Metarhizium rileyi RCEF 4871]	616	616	99%	0.0	66%	OAR02553.1
hypothetical protein V501_06700 [Pseudogymnoascus sp. VKM F-4519 (FW-2642)]	616	616	86%	0.0	75%	OAA48591.1
hypothetical protein XA68_13623 [Ophiocordyceps unilateralis]	616	616	89%	0.0	72%	KFZ07175.1
Putative Catalytic activity: AtIVD activity requires FAD as a cofactor [Torrubiella hemipterigena]	615	615	88%	0.0	72%	PFH58465.1
isovaleryl-CoA dehydrogenase [Pseudogymnoascus sp. 05NY08]	615	615	86%	0.0	73%	CEJ82834.1
Acyl-CoA dehydrogenase/oxidase [Cordyceps fumosorosea ARSEF 2679]	615	615	89%	0.0	72%	OBT77626.1
hypothetical protein VC83_07124 [Pseudogymnoascus destructans]	615	615	99%	0.0	67%	XP_018707565.1
acyl-CoA dehydrogenase domain-containing protein [Diplocarpon rosae]	615	615	89%	0.0	72%	XP_024322091.1
isovaleryl-CoA dehydrogenase [Pseudogymnoascus sp. 23342-1-I1]	615	615	84%	0.0	73%	PBP24694.1
isovaleryl-CoA dehydrogenase [Pseudogymnoascus sp. WSF 3629]	615	615	89%	0.0	72%	OBT65391.1
isovaleryl-CoA dehydrogenase [Fusarium oxysporum f. sp. lycopersici 4287]	615	615	89%	0.0	72%	OBT41881.1
	615	615	89%	0.0	72%	XP_018237377.1

FigS1. The top 100 blast results of MoIvd amino acids against all species by using the blastp program in NCBI database. All the resulting 100 blast hits were proteins from fungal species.

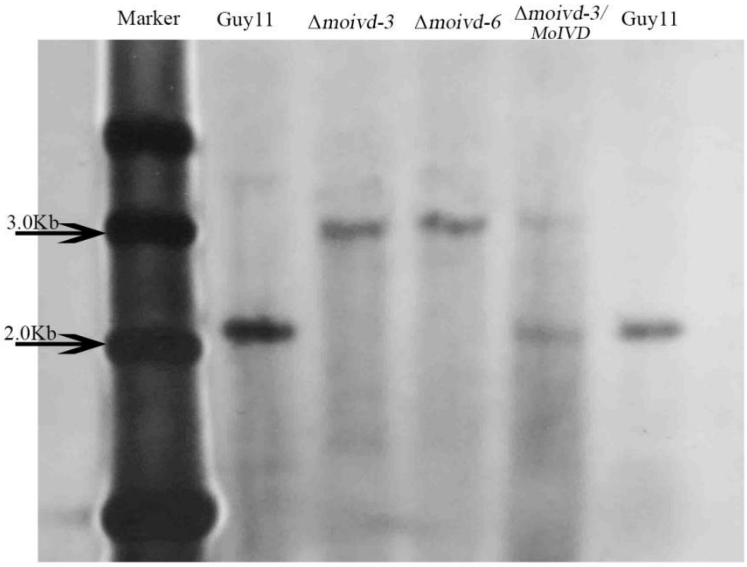

FigS2. Verification of *MoIVD* replacement by Southern blot. All the strain genomic DNA was digested with *Apa*I. A 900 bp DNA fragment in the 72 bp upstream of *MoIVD* coding region was selected as blotting probe. Consequently, a 3.1 Kb band is blotted in the Δ*moivd* mutants; the wild type Guy11 shows a 2.1 Kb band; the complementary strain Δ*moivd-3/MoIVD* is blotted with both 3.1 Kb and 2.1 Kb bands.

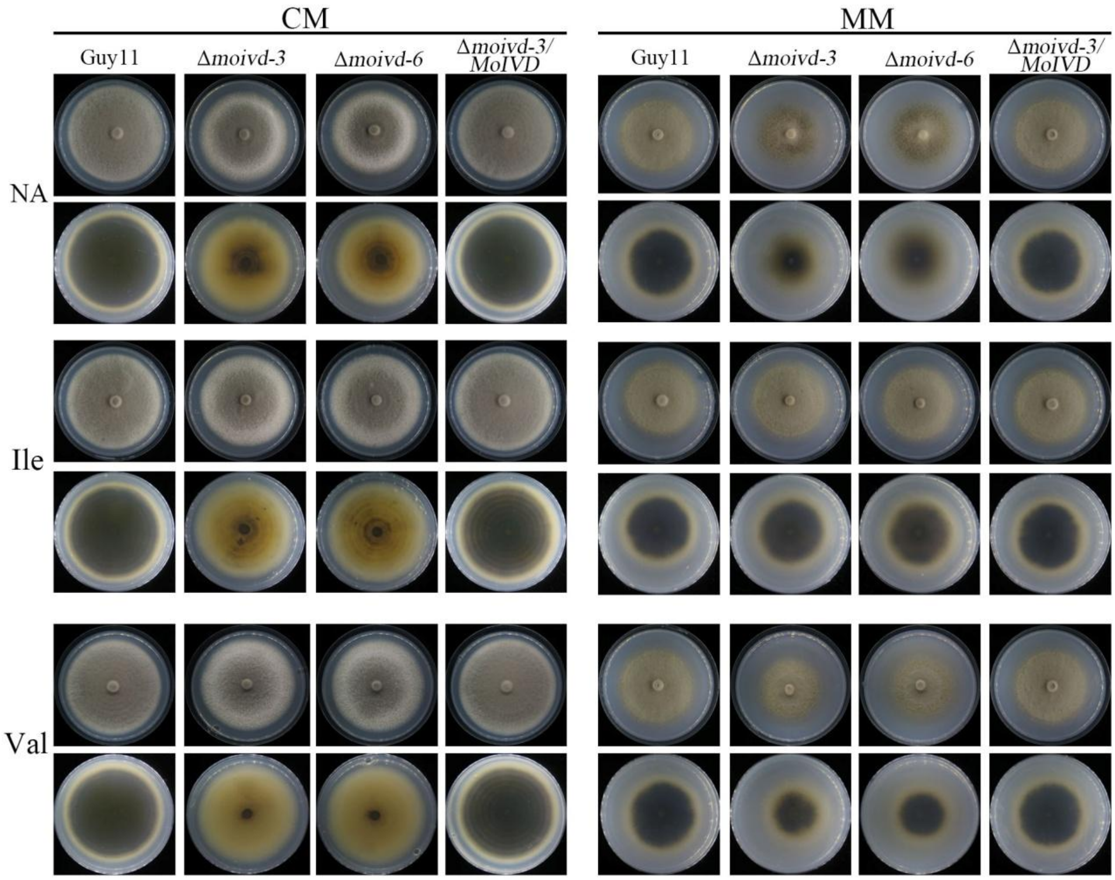

FigS3. The mutant growth phenotype tested on isoleucine (Ile) and valine (Val) containing medium (CM and MM). “NA” means the medium was not assayed with any amino acids.

(A)

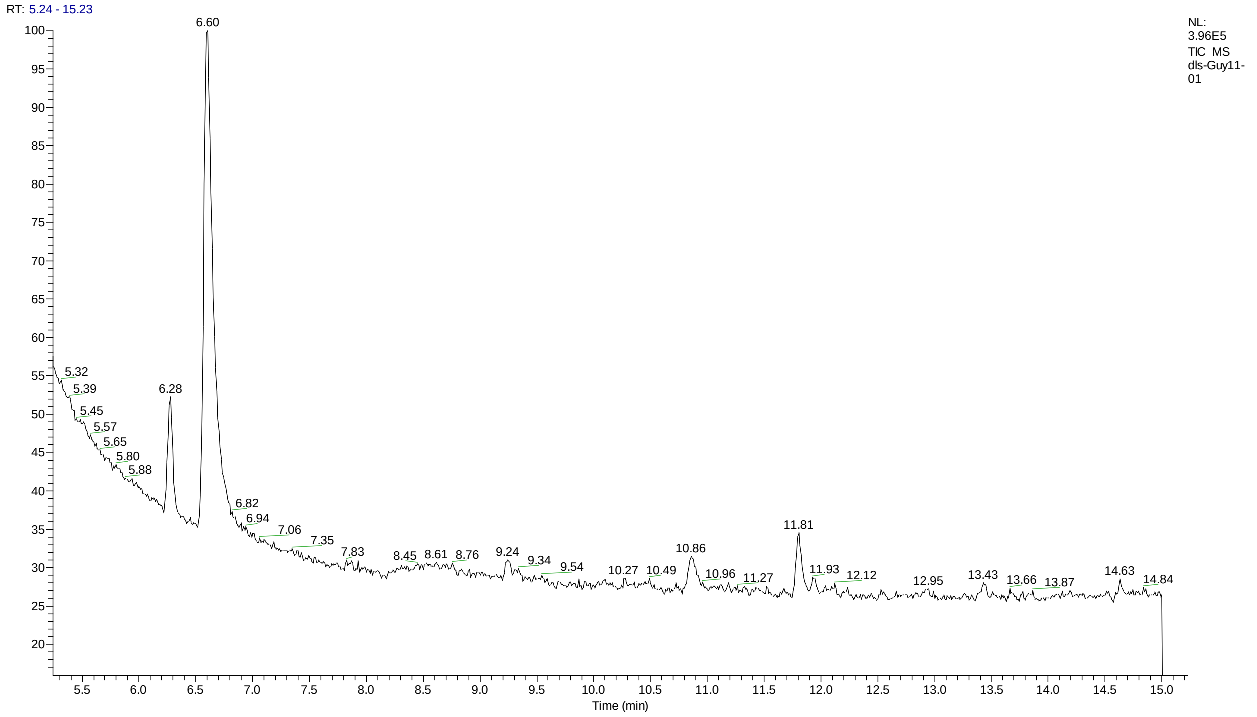
RT: 5.24 - 15.23
NL:
3.96E5
TIC MS
dls-Guy11-
01
Time (min)

(B)

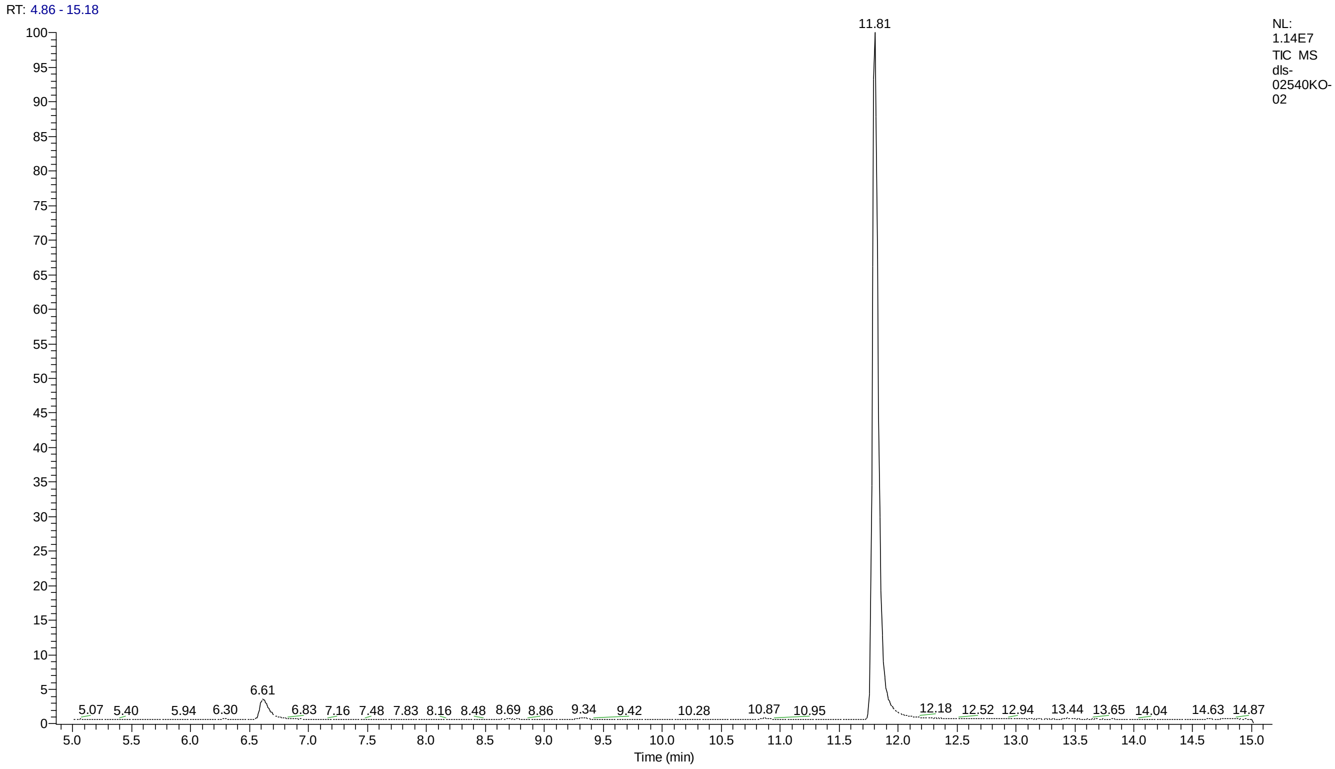
RT: 4.86 - 15.18
NL:
1.14E7
TIC MS
dls-
02540KO-
02
Time (min)

(C)

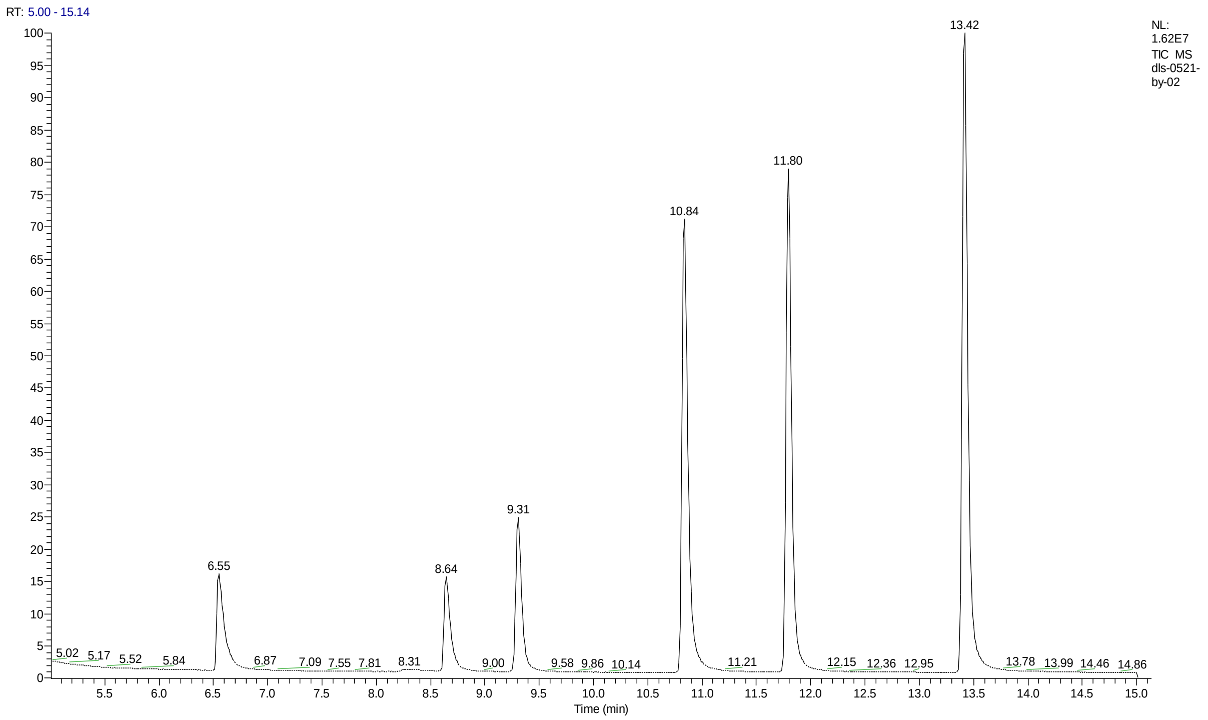

(D)

Short chain fatty acids	Guy11(μg/g)	Δ*moivd-3*(μg/g)
Acetic acid	4.85±0. 26	5.96±0. 48
Propionic acid	0.05±0. 01	0.07±0. 01
isobutyric acid	0.12±0. 02	0.36±0. 03
Butyric acid	0.08±0. 02	0.1±0.02
Isovaleric acid	0.12±0. 03	**54.09±**3. 43
Valeric acid	0.02±0. 01	0.04±0. 02

FigS4. The peak figure of short chain fatty acids in Guy11 (A), Δ*moivd-3* (B) and standard sample (C) analyzed by GC-MS assay. The real content of each fatty acid was showed in part D. A high concentration of isovaleric acid was found in Δ*moivd-3*.

(A)

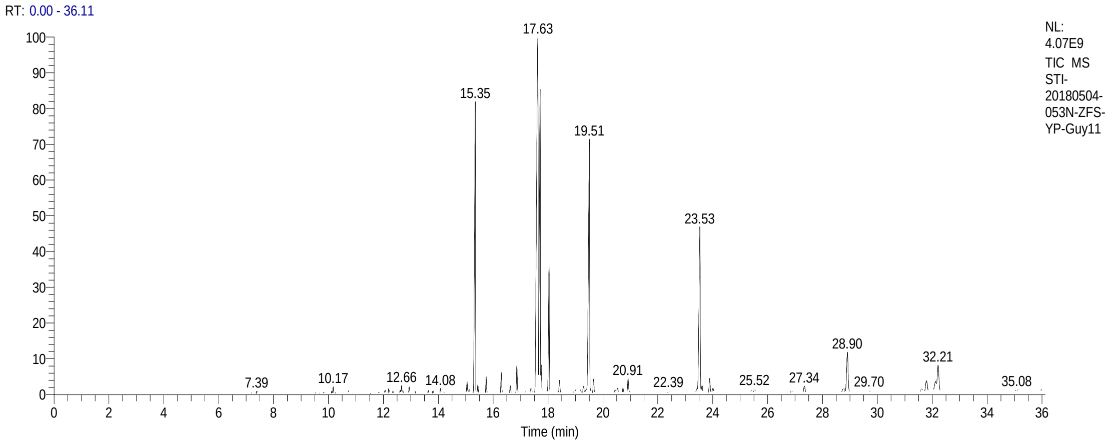

(B)

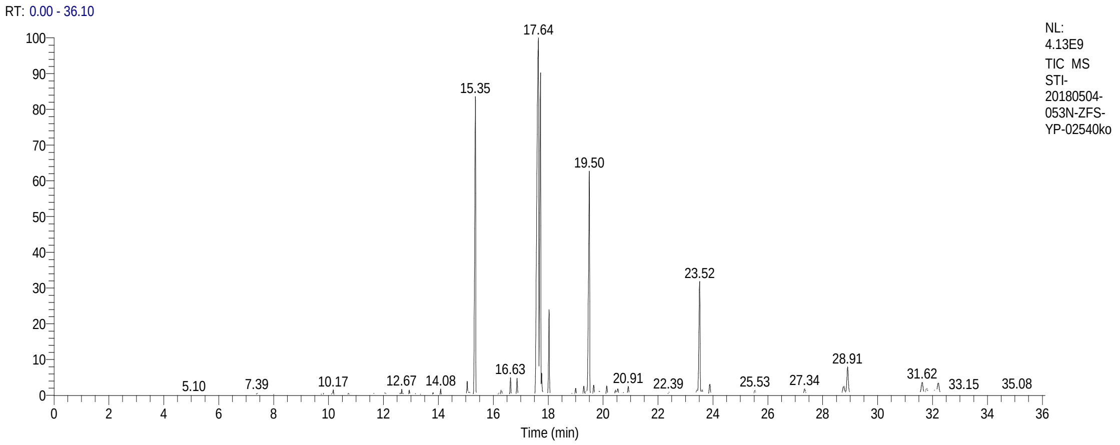

(C)

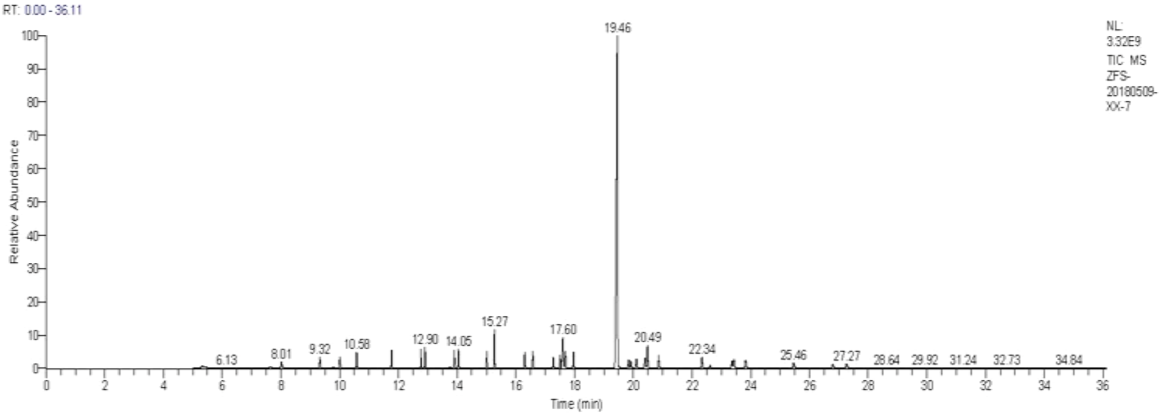

(D)

Fatty acids	Guy11(μg/g)	Δ*moivd-3* (μg/g)
C8.0	0.23±0. 05	0.44±0. 04
C10.0	0.09±0. 04	0.10±0. 03
**C11.0**	**0.012±0. 007**	**0.036±0. 015**
C12.0	0.55±0. 08	0.45±0. 16
C13.0	0.18±0. 05	0.12±0. 03
C14.0	7.03±2. 0	6.31±0. 68

C14.1	1.02±0. 34	0.75±0. 66
C15.0	6.36±1. 02	8.24±0. 77
C15.1	Undetected(<0.5)	undetected(<0.5)
C16.0	790.44±80. 05	957.73±161. 64
C16.1	25.36±5. 70	29.65±4. 4
**C17.0**	**11.16±2. 52**	**28.65±3. 13**
C17.1	5.77±1. 54	10.41±1. 53
C18.0	233.29±54. 79	158.56±27. 76
C18.1N9C	21.58±4. 65	22.92±4. 12
C18.1N9T	undetected(<0.5)	undetected(<0.5)
C18.2N6C	2248.42±371. 83	3058.68±244. 73
C18.2N6T	undetected(<0.5)	undetected(<0.5)
C18.3N3	142.33±21. 7	241.74±28. 32
C18.3N6	undetected(<0.5)	undetected(<0.5)
C20.0	27.99±3. 0	14.23±2. 05
C20.1	6.45±1. 18	6.63±0. 47
C20.2	8.67±1. 33	11.36±1. 73
**C20.3N3**	**1.39±0. 32**	**3.85±0. 60**
C20.3N6	4.39±0. 78	8.13±0. 85
**C20.4N6**	**2.90±0. 29**	**12.66±1. 17**
C20.5N3	undetected(<0.5)	undetected(<0.5)
C21.0	5.25±1. 01	6.07±0. 43
C22.0	45.24±5. 06	34.98±4. 40
C22.1N9	759.73±63. 53	445.50±35. 00
C22.2	20.02±3. 09	13.88±1. 97
C22.6N3	undetected(<0.5)	undetected(<0.5)
C23.0	16.82±2. 76	21.44±2. 07
C24.0	56.44±4. 32	42.35±4. 35
C24.1	22.82±3. 41	16.35±1. 54

FigS5. The peak figure of medium and long chain fatty acids in Guy11 (A), Δ*moivd-3* (B) and standard sample (C) analyzed by GC-MS assay. The real content of each fatty acid was showed in part D.

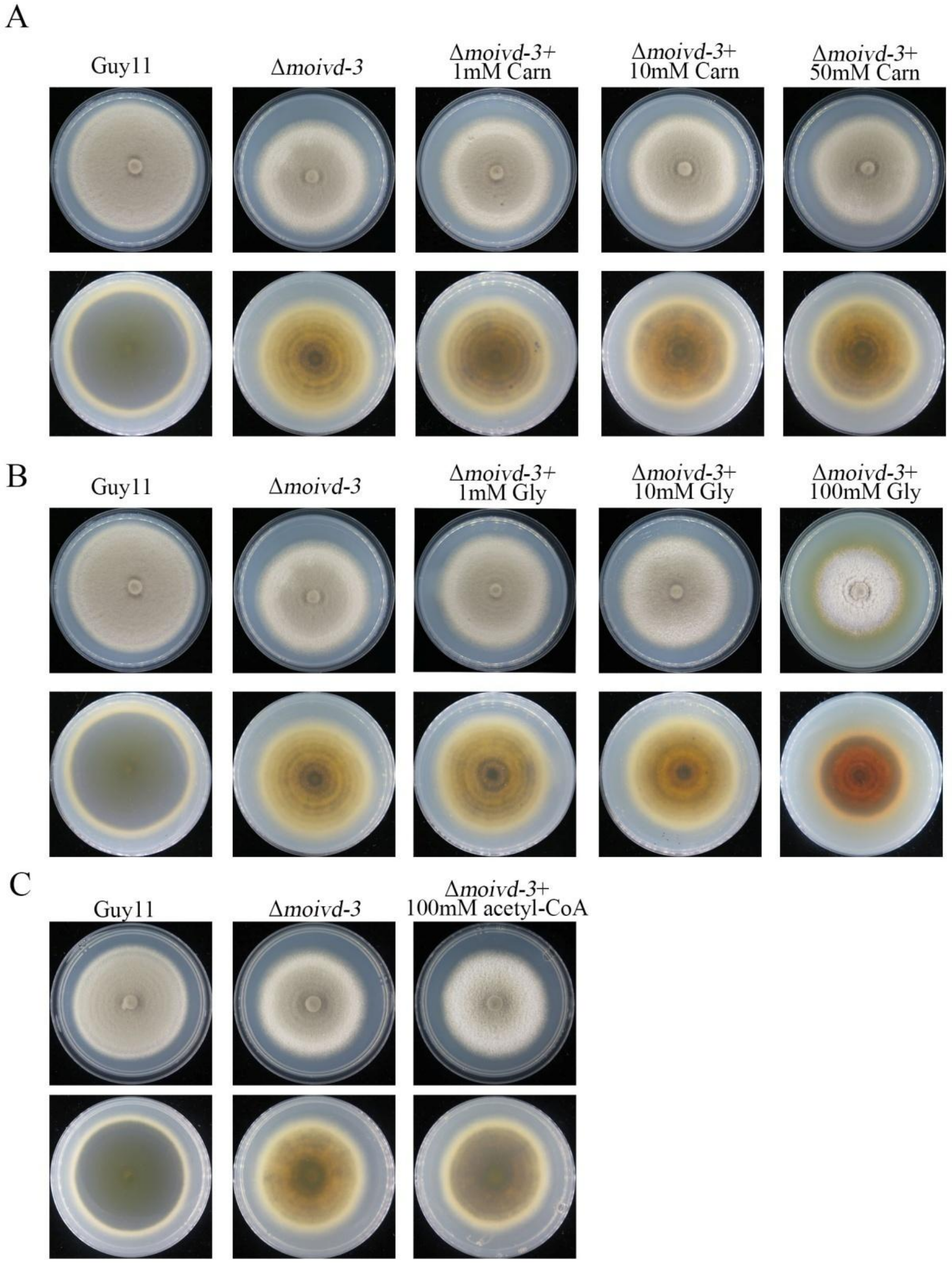

FigS6. The Δ*moivd-3* growth phenotype tested on L-carnitine (A), glycine (B) and acetyl-CoA (C) containing CM medium. Carn, L-carnitine; Gly, glycine. Δ*moivd-3* could not recover the growth phenotype when growing on CM with L-carnitine, glycine or acetyl-CoA.

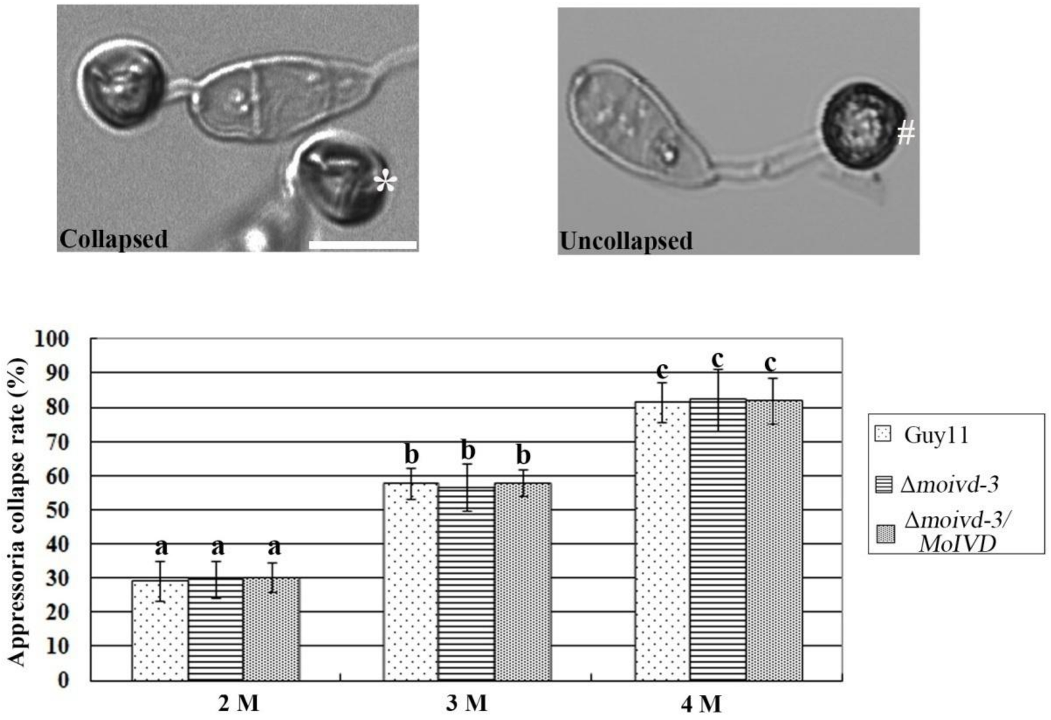

FigS7. The cytorrhysis assay testing the tutor of Δ*moivd-3* by computing collapsed appressorium rate. * indicates collapsed appressorium; # indicates uncollapsed ones. Bar=10 μm. No significant difference of appressorium collapsing rate was found between Guy11 and Δ*moivd-3* at each concentration (2 M, 3 M and 4 M) of glycerol. All these data were calculated from three independent replicates. The same lowercases ′a′ or ′b′ on the sample bar indicate no significant differences between samples. The different lowercases indicate significant differences ($P < 0.05$; t test).

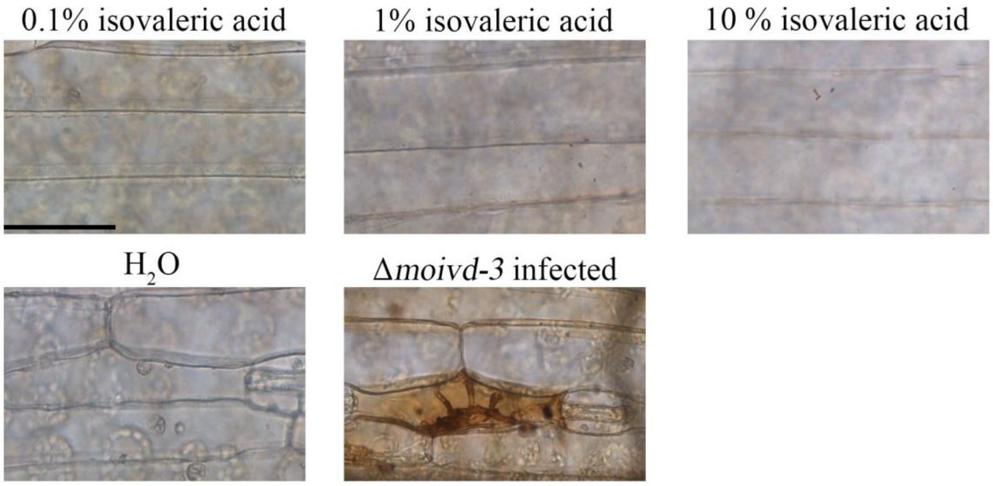

FigS8. DAB staining assay testing the ROS production in barley leaves treated by 0.1, 1% and 10% isovaleric acid. The H_2O and Δ*moivd-3* infected treatments were used as negative and positive controls, respectively. Bar=30 μm. No ROS could be induced in host cell by exogenous isovaleric acid.

(A)

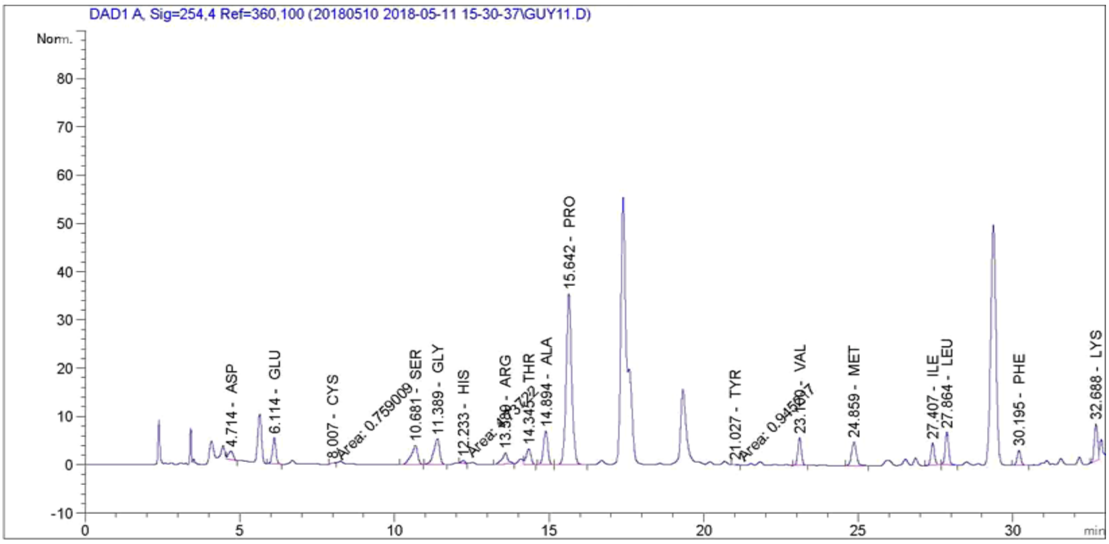
DAD1 A, Sig=254,4 Ref=360,100 (20180510 2018-05-11 15-30-37\GUY11.D)
Norm.
4.714 - ASP
6.114 - GLU
8.007 - CYS
10.681 - SER
11.389 - GLY
12.233 - HIS
ARG
THR
14.894 - ALA
15.642 - PRO
21.027 - TYR
VAL
24.859 - MET
27.407 - ILE
27.864 - LEU
30.195 - PHE
32.688 - LYS
min

(B)

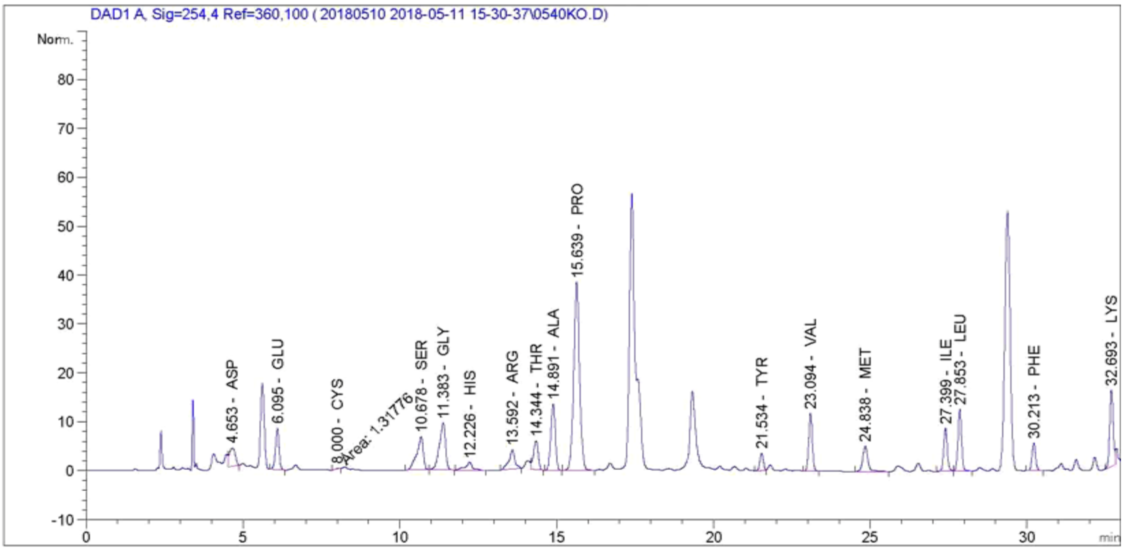
DAD1 A, Sig=254,4 Ref=360,100 (20180510 2018-05-11 15-30-37\0540KO.D)
Norm.
4.653 - ASP
6.095 - GLU
8.000 - CYS
Area: 1.31776
10.678 - SER
11.383 - GLY
12.226 - HIS
13.592 - ARG
14.344 - THR
14.891 - ALA
15.639 - PRO
21.534 - TYR
23.094 - VAL
24.838 - MET
27.399 - ILE
27.853 - LEU
30.213 - PHE
32.693 - LYS
min

(C)

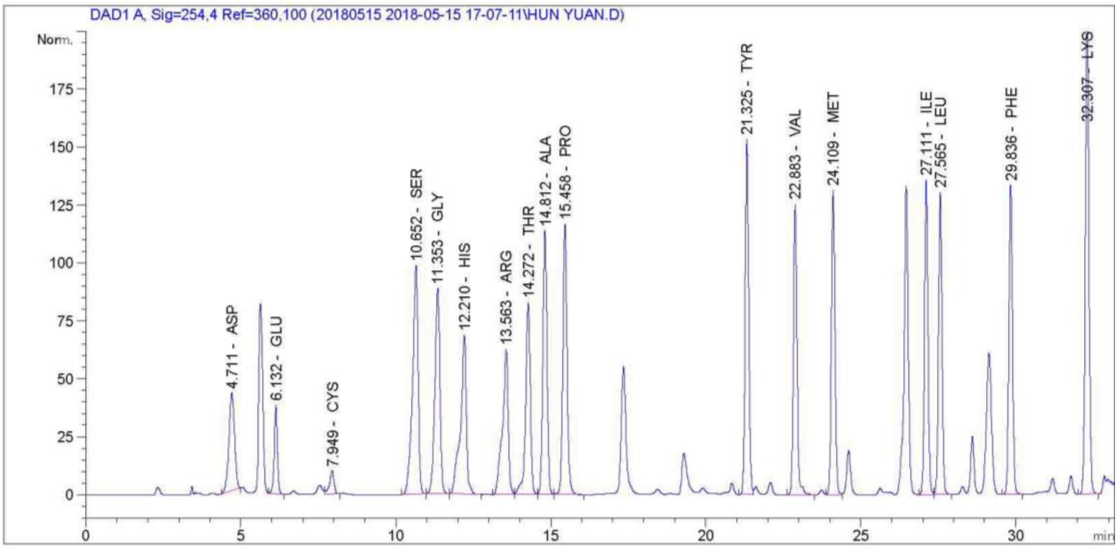
DAD1 A, Sig=254,4 Ref=360,100 (20180515 2018-05-15 17-07-11\HUN YUAN.D)
Norm.
4.711 - ASP
6.132 - GLU
7.949 - CYS
10.652 - SER
11.353 - GLY
12.210 - HIS
13.563 - ARG
14.272 - THR
14.812 - ALA
15.458 - PRO
21.325 - TYR
22.883 - VAL
24.109 - MET
27.111 - ILE
27.565 - LEU
29.836 - PHE
32.307 - LYS
min

(D)

Amino accids	Guy11(μg/g)	Δ*moivd-3*(μg/g)
Aspartic acid	0.87±0. 35	1.09±0. 20
Glutamic acid	3.28±0. 37	3.68±0. 16
Cystine	1.65±0. 33	1.62±0. 10
Serine	1.14±0. 37	1.36±0.13
Glycine	0.99±0. 24	1.12±0. 13
Histidine	0.91±0. 21	0.96±0. 07
Arginine	1.19±0. 24	1.32±0. 25
Threonine	0.78±0. 23	0.92±0. 04
Alanine	1.25±0. 29	1.48±0. 15
Proline	5.62±0. 41	5.51±0. 47
Tyrosine	0.69±0. 22	0.86±0. 06
Valine	0.97±0. 12	1.45±0. 09
Methionine	1.20±0. 19	1.15±0. 07
Isoleucine	0.85±0. 12	1.16±0. 1
Leucine	1.02±0. 11	1.50±0. 11
Phenylalanine	0.83±0. 1	0.97±0. 16
Lysine	1.25±0. 16	1.26±0. 25

FigS9. The peak figure of amino acids in Guy11 (A), Δ*moivd-3* (B) and standard sample (C) tested by HPLC assay. The real content of each amino acid was showed in part D.

Supplementary Tables

Table S1. Primers used in this study

Name	Sequence	Usage
MG02540-up-F	ATCGGCCTCTGGGTAGACTT	*MoIVD* deletion
MG02540-Hph-up-R	TTGACCTCCACTAGCTCCAGCCAAGCCGGCGGCAAGGTACATTAGAA	*MoIVD* deletion
MG02540-Hph-dw-F	GAATAGAGTAGATGCCGACCGCGGGTTGGTGATTGTCATGCACTGCT	*MoIVD* deletion
MG02540-dw-R	CTTGGCAGTATGCACCATTG	*MoIVD*deletion
MG02540-Probe-F	ATCGGCCTCTGGGTAGACTT	*MoIVD* probe
MG02540-Probe-R	ATTGACCACGGTGCTAATGG	*MoIVD* probe
MG02540-qRT-F	CAAGATGTGGATCACCAACG	*qRT*
MG02540-qRT-R	TGACGTTCTCAAGCACAAGC	*qRT*
Tublin-qRT-F	TGACAACGAGGCTCTGTACG	*qRT*
Tublin-qRT-R	CCATGAAGAAGTGCAGACGA	*qRT*
pCB1532Fadaptor-MoIVDProm-BamH1-F	CGCTCTAGAACTAGTGGATCCTCGTTGGTAGGCCAGTTCAGG	Complementation
GFPadaptor-MoIVD-Cds-EcoR1-R	GCCCTTGCTCACCATGAATTCTTGGTTGAGCGAGTACTCCCTG	*C*omplementation
pGBKT7-cds01744-EcoR1-F	ATGGCCATGGAGGCCGAATTCATGTCTGCATTGAGGATATTAGTGCC	Yeast Two-hybrid
pGBKT7-cds01744-BamH1-R	CCGCTGCAGGTCGACGGATCCTTACAATGCCCCCAGTTCCTT	Yeast Two-hybrid
pGADT7-cds02540-EcoR1-F	GCCATGGAGGCCAGTGAATTCATGGCTAGCATGCGAGCAGT	Yeast Two-hybrid
pGADT7-cds02540-BamH1-R	CAGCTCGAGCTCGATGGATCCTCATTGGTTGAGCGAGTACTCCC	Yeast Two-hybrid
pGBKT7-cds01719-EcoR1-F	ATGGCCATGGAGGCCGAATTCATGCTCTCGGCAGCACGAAGA	Yeast Two-hybrid
pGBKT7-cds01719-BamH1-R	CCGCTGCAGGTCGACGGATCCTCATGACTTGCCGAGCTTCT	Yeast Two-hybrid
pKNT-NYFP-02540-Prom- KpnI-F	AGGGAACAAAAGCTGGGTACC TCGTTGGTAGGCCAGTTCAGGTT	BIFC
PKNT-NYFP-02540-HindIII –R	CGTGGCGATGGAGCGAAGCTTTTGGTTGAGCGAGTACTCCCTGTT	BIFC
Pcx62-CYFP-01744-Prom- KpnI-F	AGGGAACAAAAGCTGGGTACC ATGTCTGCATTGAGGATATTAGT	BIFC
Pcx62-CYFP-01744-HindIII –R	CTTGCAGGCCGGGCGAAGCTTCAATGCCCCCAGTTCCTTGAG	BIFC
Pcx62-CYFP-01719-Prom- KpnI-F	AGGGAACAAAAGCTGGGTACCATGCTCTCGGCAGCACGAAGA	BIFC

Table S2. The conidial and appressorial phenotype of the Δ*moivd* mutants

Strains	**Conidial Germination(%)[a]**			**Appressorium formation(%)[b]**	
	**2h**	**4h**	**6h**	**6h**	**8h**
Guy11	85.3±2.5A[c]	94.7±2.5A	97.7±2.1 A	81.7±8.1 A	90.7±6.0 A
Δ*moivd-3*	84.7±4.0A	93.7±4.0A	98.7±1.5 A	80.3±4.5A	91.3±7.0 A
Δ*moivd-6*	85.0±3.6A	93.3±2.5A	98.0±3.5 A	82.0±6.0 A	91.7±8.1A
Δ*moivd-3/MoIVD*	84.3±2.1A	94.3±2.1A	97.7±3.2 A	982.7±7.8 A	91.3±6.5 A

a. Percentage of germinated conidia on plastic hydrophic surface incubation for 2h, 4h and 6h at 25℃.

b. Percentage of appressoria formation on plastic hydrophic surface incubation for for 6h and 8 h at 25℃.

c. Data were calculated from three independent experiments conducted in triplicates. Same capital letters indicated no significant difference at P-value of 0.01 (t test).
